# Interplay between the Molecular Structure of Lignin and the Degradation Process of PLA/Lignin Materials

**DOI:** 10.1021/acs.biomac.5c02467

**Published:** 2026-04-14

**Authors:** Paula Pou I Rodríguez, Karin Odelius, Minna Hakkarainen

**Affiliations:** † Wallenberg Wood Science Center (WWSC), KTH Royal Institute of Technology, Teknikringen 56, Stockholm 100 44, Sweden; ‡ Department of Fiber and Polymer Technology, KTH Royal Institute of Technology, Teknikringen 58, Stockholm 100 44, Sweden

## Abstract

Blending lignin in polylactide (PLA) could simultaneously promote lignin valorization and tackle the limitations of PLA. Still, the lack of compatibility between lignin and PLA typically requires chemical modification of lignin’s hydroxyl groups, which could affect the already slow biodegradation rate. Here, the impact of the molecular structure of different lignin types on the properties and degradation processes of PLA/lignin films was investigated. Alkaline lignin and Kraft lignin were blended with PLA as received and after acetylation and/or fractionation. The PLA/lignin materials were aged under hydrolytic and simulated industrial composting conditions. During hydrolytic degradation, PLA/lignin materials with acetylated lignin showed an accelerated degradation rate during the early stages, as a consequence of lignin deacetylation and the release of acetic acid that catalyzed ester hydrolysis. Our results demonstrate that the molecular structure and weight of lignin influence both the physicochemical properties and morphology of the films, as well as the subsequent degradation process.

## Graphical abstract

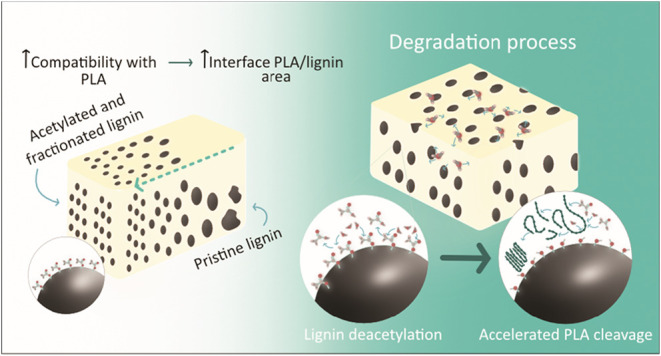

## Introduction

Increased concerns over the negative environmental consequences posed by fossil-based materials have led to a shift toward a more sustainable mindset. This is observable in the increased amount of political actions toward the implementation of greener practices, the use of renewable resources, and the development of a more circular economy.^1–3^ A considerable amount of research has been devoted to the development of sustainable materials from biobased origins. Lignocellulose is the main source of biomass, corresponding to around 60% of biogenic carbon in the biosphere. It is mainly composed of a mixture of cellulose, hemicellulose, and lignin.^4^ Lignin has emerged as a potential source for new chemicals, materials, and fuel, as it is the most abundant aromatic biopolymer. The molecular structure of lignin is dominated by three building blocks, namely, coumaryl, coniferyl, and hydroxyphenyl, interlinked through mainly aryl ether (β-O-4), phenyl coumaran (β-5), and resinol (β-β) bonds. There is currently great interest in lignin valorization to create new innovative materials by utilizing rich molecular structures with phenolic, carboxylic, and aliphatic hydroxyls, aromatic moieties, and methoxy groups.^5–8^ In addition, lignin has the ability to bestow antioxidant and antibacterial properties and UV-protection to create materials with value-added properties.^9–11^ Incorporation of lignin in polymer matrices has potential advantages, such as increased biobased content, mechanical reinforcement, and lower cost for the final product.^12,13^ There are four main technical lignins, produced by different pulping methods, i.e., Kraft, lignosulfonate, soda, and organosolv lignin.^12^ The wide range of physicochemical properties, originating from the heterogeneity of the molecular structure, which depends on the biomass source and pulping method, has however complicated the introduction of lignin-derived products into the market.^14^

Polylactide (PLA) is one of the most important synthetic biobased polymer options available commercially. It is biodegradable and biocompatible with comparable mechanical properties and processability to conventional plastics.^15^ Despite that, the expected market growth of PLA has been limited due to its higher price compared to many common plastics and in some aspects limited properties such as brittleness, slow crystallization rate, slow biodegradation rate, and relatively low thermal resistance.^16,17^ The convergence between the need to overcome the drawbacks of PLA and the potential value-added properties of lignin drives the development of PLA/lignin materials and contributes to reaching a more circular economy model. However, the differences in the molecular structure with respect to PLA, including the high number of polar hydroxyl groups, strong hydrogen bonding, and π–π stacking within lignin, typically cause poor compatibility and aggregation.^18,19^ This is also anticipated from the differences in the Hansen solubility parameters.^20,21^ Lignin aggregation in the blends negatively affects important properties, such as thermal stability, tensile strength, and elongation at break. Due to the aggregation, property improvement is typically only achieved when lignin is added in very small quantities.^11,22^ Chemical modification focusing on hydroxyl substitution is the primary strategy employed to develop high quality PLA/lignin materials.^13,19,23^ Here, lignin acetylation has been proven as a simple method to enhance the compatibility between PLA and lignin.^11,24,25^ On the other hand, the hydroxyl content, especially aromatic hydroxyls, could increase the biodegradability of lignin materials.^5,26^ Several papers have investigated how lignin affects the physicochemical properties and morphology of PLA/lignin materials. However, a more complete molecular-level characterization of these materials is missing. In addition, the impact of the different structural features of lignin on the properties and degradation process of both PLA and lignin in different environments has been overlooked.^24,27,28^ This has high importance for implementation of PLA/lignin materials in e.g., biomedical, packaging and agricultural products.^29,30^ Here, we designed a selection of lignin materials, including Kraft and alkaline lignin as well as fractionated and/or acetylated lignins, aiming to reveal the influence of different molecular structures and molecular weights on the physicochemical properties and degradation behavior of PLA/lignin materials under hydrolytic and simulated industrial composting conditions. Understanding the degradation mechanisms and rate of PLA/lignin materials under different conditions defines the potential application areas and further benefits the implementation of processes such as chemical recycling,^31^ targeted degradation in a specific environment,^32^ and production of value-added materials such as PLA/lignin blend-derived carbon fibers.^33^ The molecular heterogeneity of lignin is anticipated to influence the interactions and compatibility with PLA. We also hypothesized that acetylation of lignin’s abundant hydroxyl groups would both improve the compatibility and influence the degradation process. Deacetylation is the rate-determining step during the biodegradation of cellulose acetate.^34^ In the same way, acetylation could prohibit the subsequent biodegradation of lignin. However, if deacetylation takes place under the degradation conditions, this could promote the degradation process due to the release of acetic acid, which could catalyze ester hydrolysis and accelerate the degradation rate of PLA.^35^ Multiple tools were utilized to monitor in detail the physicochemical properties and structural changes taking place in both PLA and lignin during the degradation process.

## Experimental Section

### Materials

Polylactide (PLA) IngeoTM 4043D grade was obtained from NatureWorks, with 4.3% of d-isomer as determined by others.^36^ Alkaline lignin with a low degree of sulfonate groups (AL) (4% sulfur, water-soluble), acetic anhydride (≥99%), dimethyl sulfoxide-D6, chloroform-D1, and Plate Count Agar (Tryptone Glucose Yeast) were purchased from Sigma-Aldrich. Formic acid 98% was bought from Honeywell Fluka. Softwood Kraft lignin UPM BIOPIVATM 100 (KL) was purchased from UPM Biochemicals, Finland. Ethanol, dichloromethane (DCM), and industrial sand were purchased from VWR Chemicals. For ^31^P NMR analysis, chromium­(III) 2,4-pentanedionate (Cr­(acac)_3_; 97%), 2-chloro-4,4,5,5-tetramethyl-1,3,2-dioxaphospholane (TMDP; 95%), and pyridine (≥99.0%) were acquired from Sigma-Aldrich. *N*-Hydroxy-5-norbornene-2,3-dicarboxylic acid imide (NHND; >99.0%) was purchased from Tokyo Chemical Industry U.K. Ltd. Soil was purchased from Gardol Toppdress, and compost was retrieved from a local garden.

### Microwave-Assisted Fractionation and Acetylation of Lignin

To reduce the molecular weight and dispersity, KL was fractionated by microwave processing in 2% formic acid in ethanol according to a previous procedure.^37^ In short, 0.5 g of KL was mixed with 18 mL of ethanol and 2 mL of formic acid in high-pressure Teflon vessels and heated to 160 °C with a ramp time of 20 min in a flexiWAVE MA186–001 microwave. A maximum power of 800 W was used. After the reaction, the liquid fraction was collected by filtration and dried for 48 h in a vacuum oven. Lignin esterification is a common practice to increase the compatibility with thermoplastic polymers. In this work, AL, KL, and KL-Fract were acetylated by a microwave-assisted reaction with acetic anhydride without other additional solvents or catalysts according to a previously reported procedure.^11^ In summary, lignin and acetic anhydride were introduced in high-pressure Teflon vessels (1:5 w/w lignin:solvent ratio) and heated in a flexiWAVE MA186–001 microwave. A ramp time of 5 min to 130 °C and a 10 min isotherm were used with a maximum power of 800 W. After the reaction, the contents were stirred for 24 h in distilled water and vacuum-filtrated before subsequent drying in a vacuum oven at 60 °C.

### Preparation of PLA/Lignin Films

Solvent casting was utilized to produce thin films of PLA/lignin and neat PLA. All the blend films were cast with the same ratio of PLA and lignin (7/3 w/w) to ensure good mechanical integrity of the final films while incorporating enough lignin to be able to evaluate its influence on the degradation process. All films were cast on 22 cm diameter glass Petri dishes, using a total of 3 g of the material for each film and a concentration of 0.1 g/mL of DCM. First, PLA was dissolved in DCM for 1 h at room temperature, and then lignin was added during continuous magnetic stirring to ensure good mixing. Afterward, the solution was cast, covered with perforated aluminum foil, and kept in a fume hood for 24 h, after which the films were further dried in a vacuum oven for a minimum of 48 h.

### Hydrolytic Degradation

The hydrolytic degradation of the films was investigated for a period of 30 days in distilled water at 60 °C, which is similar to the temperature during simulated industrial composting. First, the films were cut into rectangular shapes of 5 cm × 0.5 cm and placed in 100 mL glass vials. Then, the vials were filled with 20 mL of distilled water and stacked horizontally in a Venticell 111 ECO oven to ensure complete sample immersion. A set of 3 replicates for each type of film and testing time were prepared. The vials were taken out after the predetermined testing times (1, 3, 5, 10, 15, 20, or 30 days). The remaining films were weighed, dried in a vacuum oven for a minimum of 48 h, and weighed again before characterization. Three parallel experiments were performed to simulate the potential catalytic effect of released acetic acid on the hydrolytic degradation of PLA. In all three experiments, 5 × 0.5 cm rectangular films were placed in 10 mL of medium for 3 days at 60 °C. In the first experiment, neat PLA was placed in distilled water (named PLA/neutral), in the second experiment, neat PLA was placed in acidic water containing acetic acid (pH 3) (named PLA/acid), and in the third experiment, acetic acid was embedded in PLA films during the casting process. The acetic acid concentration was adjusted to the theoretical amount of acetic acid release from KL-Acet by deacetylation (the theoretical maximum amount of acetic acid release from PLA/KL-Acet films is 0.098 g of acetic acid/g of PLA). The vials were taken out after 24 and 72 h, and the samples were dried in a vacuum oven for a minimum of 48 h.

#### Water Absorption and Weight Loss

The water absorbed (% *w*_abs_) and weight loss % (% *w*_loss_) of the films after different hydrolysis times were calculated. Excess water was removed before weighing with tissue paper. The amount of absorbed water was calculated using eq 1, where *w*_d_ is the dry weight after hydrolysis, and *w*_w_ is the wet weight after the same hydrolysis time. The % *w*_loss_ was calculated following eq 2, where *w*_o_ is the initial weight. The values for the 3 replicates were averaged, and the standard deviation was included as the error bar.

1 $$\%\;w_{\mathrm{abs}}=100\times \frac{w_{w}-w_{d}}{w_{d}}$$

2 $$\%\;w_{\mathrm{loss}}=\frac{w_{o}-w_{d}}{w_{o}}\times 100$$

#### pH Measurements

To monitor the pH evolution in the aging medium, the pH of the aqueous medium after each aging period was determined by a Mettler Toledo FiveEasy Plus using a LE438 sensor. The values for the 3 replicates were averaged, and the standard deviation was included as the error bar.

### Simulated Industrial Composting

Simulated compost experiments were performed to evaluate the combined effect of humidity, heat, and microorganisms on PLA/lignin samples. The compost was prepared by mixing soil and compost at a 1/9 w/w ratio. To ensure microbial activity in the compost, the following protocol was implemented: First, 1 g of compost was mixed with 10 mL of Milli-Q water in a vortex for 3 min. The liquid fraction was serially diluted 6 times (from 10^–1^ to 10^–6^ g/mL) with sterile water, and each concentration was transferred into a different agar plate. The plates were left to incubate at 37 and 55 °C for 72 h, after which the presence of colonies at concentrations 10^–1^ to 10^–4^ at both temperatures indicated microbial activity in the compost. Closed 1 L glass wide-neck bottles were used as reactors for simulated composting. First, 150 g of sand was placed at the bottom of each jar, followed by the introduction of 150 g of compost mixed with 2 g of 0.5 × 5 cm shaped samples, and finally, this was topped with 100 g of sand. The tightly closed jars were placed in a Venticell 111 ECO oven. For each film type, 3 jars were prepared. The temperature was set at 60 °C for a period of 105 days. At day 63, one of the jars for each film type was opened, and a small fraction of the aged films was recovered; moreover, at day 105, all remaining samples were collected, washed, and dried before characterization. The values for the 3 replicates were averaged, and the standard deviation was included as the error bar.

### Characterization of PLA, Lignin Materials, and PLA/Lignin Films Including Aged Films

The influence of different lignin fractions on the properties and degradation behavior of PLA/lignin films under different conditions was investigated by multiple characterization techniques.

#### Fourier Transform Infrared (FTIR) Spectroscopy

The functional groups of nondegraded and degraded PLA/lignin films were analyzed on a PerkinElmer Spectrum 100 FTIR spectrometer (PerkinElmer Inc., USA). All spectra were recorded in the range of 4000–600 cm^–1^, using 32 scans at a resolution of 4 cm^–1^.

#### 2D NMR Heteronuclear Single Quantum Coherence (HSQC) Spectroscopy

To investigate the evolution of the molecular structure of lignin during hydrolytic degradation, a semiquantitative analysis of the interunit linkages of lignin was done via 2D NMR HSQC. PLA/lignin samples before and after hydrolytic degradation were cut into small pieces and submerged in 0.6 mL of DMSO-*d*_6_ with a target lignin weight of approximately 80 mg. The spectra were acquired using the pulse sequence “hsqcetgpsi2”, 128 scans, a relaxation delay of 2 s, and an acquisition time of 0.1280 s at a temperature of 298 K with a 400 MHz Bruker Advanced III HD spectrometer. The spectral window used was 170 ppm for F1 and 10 ppm for F2 by using 1024 × 256 increments. Data were processed using MestReNova software (Mestrelab Research SL), and a manual phase correction was performed on both dimensions, followed by the reference adjustment at 2.5/39.5 ppm of the DMSO-*d*_6_ solvent. The aromatic region C_2_ of the guaiacyl lignin unit was used as a standard to calculate the relative abundance of interunit linkages of lignin. Tables S1 and S4 present the assignment of each spectral region.

#### Size Exclusion Chromatography (SEC)

The molecular weight and dispersity (Đ) of both PLA and lignin originally and after extraction from the nondegraded and degraded PLA/lignin film samples were investigated. The molecular weight of PLA and lignin components was determined separately. First, the DMSO-soluble fraction in PLA/lignin films was dissolved in DMSO/0.5 w/w% LiBr (lithium bromide) by submerging the samples in the solvent at a target concentration of 5 mg/mL lignin. Due to the insolubility of PLA in DMSO, the soluble fraction was assumed to mainly consist of lignin. A SECurity 1260 Infinity GPC System (Polymer Standards Service) was used. The instrument was operated at 60 °C and eluted with dimethyl sulfoxide (DMSO) with a 0.5% (w/w) LiBr solution at a flow rate of 0.5 mL min^–1^. Narrow dispersity Pullulan standards in the molecular weight range of 342–7,08,000 Da were used for calibration, and a RI detector Agilent RID G1362A was used. WinGPC UniChrom software was used to calculate the molecular weight dispersion parameters. After the non-DMSO-soluble sample was rinsed with distilled water and dried in a vacuum oven for a minimum of 48 h, the sample was submerged in chloroform at a target concentration of 6 mg/mL. The chloroform-soluble sample mainly consisted of PLA with traces of chloroform-soluble products from the lignin. A Malvern GPCMAX equipped with an autosampler and three PLgel 5 μm columns was utilized with chloroform as an eluent at a flow rate of 0.5 mL/min and an RI detector. Narrow dispersity polystyrene standards in the range of 1200–4,00,000 g/mol were utilized to create the calibration curve. OmniSEC 5.10 software was used to calculate the molecular weight distribution parameters. The values for the 3 replicates were averaged, and standard deviations were included as error bars.

#### Differential Scanning Calorimetry (DSC)

A DSC1 STARe System (Mettler-Toledo AG, Greifensee, Switzerland) was used to evaluate the thermal transitions and degree of crystallinity (*X*_c_) of nondegraded and hydrolytically degraded PLA/lignin samples. Between 5 and 10 mg of each sample was placed in 100 μL aluminum cups and sealed with a perforated lid. All samples were submitted to a set of two heating–cooling cycles starting from 30 to 200 °C, kept isothermally at 200 °C for 3 min, cooled to −20 °C, and kept at −20 °C for 3 min, followed by subsequently heating to 200 °C. The first heating scan was used to evaluate the cold crystallization and melting characteristics, and the second to determine the glass transition temperature (*T*_g_). The heating rate was 10 °C/min, and the experiments were performed under a N_2_ atmosphere at a flow rate of 50 mL/min. The analysis of the data was done on Mettler STARe Evaluation software. Equation 3 was used to calculate the degree of crystallinity (*X*_c_), where Δ*H*_m_ is the enthalpy of melting, Δ*H*_cc_ is the enthalpy of cold crystallization, *w* is the weight fraction of PLA in the film, and Δ*H*_m_^o^ is the melting enthalpy of 100% crystalline PLA (93 J/g).^38^ For PLA/AL, from day 1, *w* is assumed to be 100% PLA due to AL dissolution in water. For the rest of the samples, the ratio between PLA and lignin is assumed to be constant throughout the degradation. This could cause some overestimation of *X*_c_ if the weight loss is caused mainly by dissolution of PLA oligomers. The values for the 3 replicates were averaged, and the standard deviation was included as the error bar.

3 $$X_{c}=\left(\frac{{\Delta H}_{m}-{\Delta H}_{\mathrm{cc}}}{w^{*}{\Delta H}_{m}^{o}}\right)\times 100$$

#### Thermogravimetric Analysis (TGA)

The thermal stability of nondegraded and hydrolytically degraded PLA/lignin samples was evaluated using a Mettler-Toledo TGA/SDTA 851e instrument (Mettler-Toledo AG, Greifensee, Switzerland). 5–10 mg of each sample was placed in 70 μL alumina cups and heated from 30 to 800 °C at 10 °C/min in a N_2_ atmosphere at a flow rate of 50 mL/min. The values for the 3 replicates were averaged, and the standard deviation was included as the error bar.

#### Field Emission Scanning Electron Microscopy (FE-SEM)

Images of the surface morphology and cross section of the films were taken before and after hydrolytic degradation. A Hitachi S-4800 ultrahigh-resolution field emission scanning electron microscope (FE-SEM) (Hitachi Ltd., Tokyo, Japan) was used at a 1 kV acceleration voltage for all images. For each sample, both sides of the film were visualized. The samples were sputter-coated with a platinum/palladium (Pt/Pd) coater target using a Cressington 208HR Sputter Coater (Cressington Scientific Instruments Ltd., Watford, U.K.) for 30 s (around 3 nm thickness). Lignin aggregate diameter size was calculated with ImageJ.

#### Particle Size Distribution in Solvent

Particle size distribution of all lignin materials in DCM was measured using a Malvern Panalytical Mastersizer 3000+ Ultra using the disperser in the Hydro SV mode. The background was measured with DCM, and the sample concentration was set to 0.3 mg lignin/mL of solvent (DCM) while 500 rpm was used during the measurement. All solutions were stirred for 30 min prior to the measurement, and 3 measurements were performed for each sample. The 3 sets of data were averaged, and the standard deviation was calculated.

#### Contact Angle

The effect of lignin on the surface hydrophobicity of PLA/lignin films was assessed by static water contact angle (WCA) measurements. The measurement was performed with an Optical tensiometer, Theta Lite from Biolin Scientific. Water droplets of 4 μL were dispensed on the film surface, and at least 3 measurements were performed for each sample replicate at different spots. The values for the 3 replicates were averaged, and the standard deviation was included as the error bar.

## Results and Discussion

PLA/lignin films were produced with Kraft or alkaline lignin, including acetylated or fractionated variants, to evaluate the influence of different lignin types on the properties and degradation behavior of PLA/lignin blends under hydrolytic and simulated composting conditions.

### Characterization of Original Lignin Materials, PLA, and PLA/Lignin Films

The original physicochemical properties of different lignins (Table 1) and the properties of neat PLA and PLA/lignin films (Figure 1) were first characterized.

**Figure 1:**
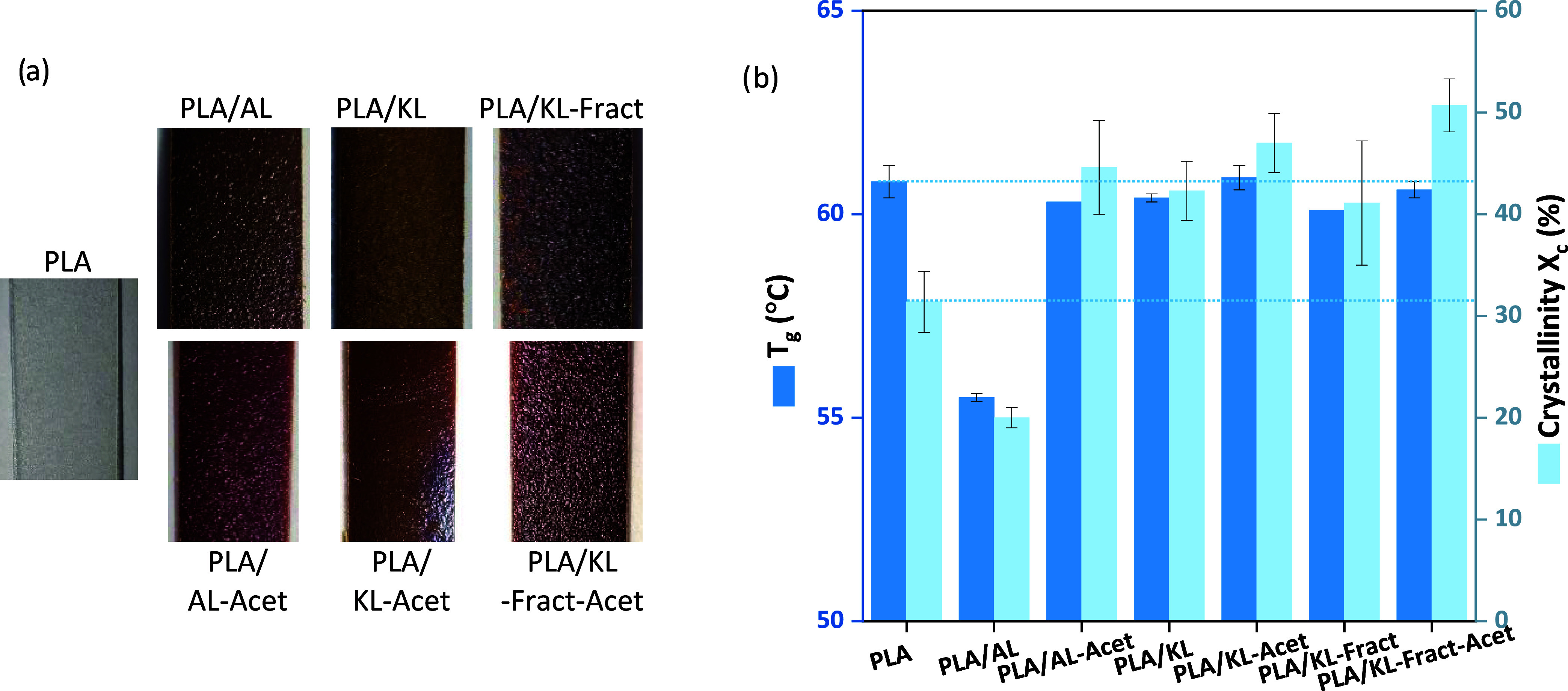
(a) Photographs showing the top surface of PLA and PLA/lignin films, and (b) glass transition temperature (*T*_g_) and degree of crystallinity (*X*_c_).

**Table 1:** Description of Different Lignin Materials Used in This Work, Including Number Average Molecular Weight (*M_n_*), Weight Average Molecular Weight (*M*_w_), Hydroxyl (OH) Concentration, Degree of Substitution (DS) of Hydroxyl Groups after Acetylation, and Yield of Fractionated and/or Acetylated Products

sample code	lignin type	fractionation/modification	*M_n_* (g/mol)	*M*_w_ (g/mol)	mmol OH/g lignin^a^	DS (%)^a^	yield (%)
AL	alkaline		1500 ± 300	8600 ± 600			
AL-Acet	alkaline	-/acetylation	1200 ± 200	13,700 ± 1400	0.8 ± 0.2	^b^	65 ± 5
KL	Kraft		1000 ± 100	7100 ± 100	5.1 ± 0.2		
KL-Acet	Kraft	-/acetylation	1100 ± 100	11,600 ± 1900	1.2 ± 0.1	76.1	87 ± 11
KL-Fract	Kraft	fractionation	800 ± 50	2400 ± 200	5.5 ± 0.5		40 ± 4
KL-Fract-Acet	Kraft	fractionation/acetylation	800 ± 90	3200 ± 200	1.3 ± 0.2	76.8	89 ± 7

a Values determined with ^31^Phosphorus-NMR (^31^P NMR): Spectra are shown in Figure S1, and the experimental procedure can be found in the SI.

b OH group quantification and DS could not be calculated due to the insolubility of the original AL in the ^31^P NMR solvent system.

All lignin samples (AL, AL-Acet, KL, KL-Acet, KL-Frac, and KL-Fract-Acet) were carefully characterized, and their molecular weight and molecular structure, including quantification of different OH groups, yield after fractionation/acetylation, and DS of acetylation were determined (Table 1). The yield provided for KL-Fract represents the lignin fraction soluble in ethanol during microwave fractionation, while the yields of acetylated KL-Acet and KL-Fract-Acet are above 85%. The somewhat lower yield of AL-Acet (65%) is explained by dissolution of water-soluble fractions during the cleaning step. The successful acetylation and fractionation of KL and AL was confirmed by ^31^P NMR and SEC (Figure S1 and Table 1). AL and KL exhibited similar molecular weights in agreement with previously reported values.^23^ The quantity of OH groups in KL also agreed with previously reported values.^37^ For KL-Fract, a pronounced decrease of *M*_w_ with respect to *M_n_* was observed, confirming the extraction of lower-molecular-weight fractions during microwave processing.^37^ A small increase in the amount of OH groups was also observed in comparison to KL, which correlates with the higher number of OH groups expected in the lower-molecular-weight lignin fractions.^39^ Both acetylated Kraft lignins portrayed similar DS and a lower amount of OH groups compared to the respective non-modified lignin. An increase in *M*_w_ for all acetylated lignins compared to the non-modified material was also observed, due to the introduction of acetate groups.^40^ The large increase in *M*_w_ could further indicate the presence of higher-molecular-weight species as a consequence of lignin condensation reactions during acetylation,^41^ while the absence of significant depolymerization reactions is supported by the fairly constant *M_n_*.

To determine the molecular weight of PLA in the PLA/lignin films after solution casting, the molecular weight and dispersity of the chloroform-soluble part (predominantly PLA) of the films were determined, as shown in Table S2. These values were used as reference molecular weights for original non-aged samples during the degradation experiments. PLA in all PLA/lignin films had lower *M_n_*, *M*_w_, and *M*_p_ (molecular weight at the peak) compared to neat PLA. Depending on lignin type, the *M*_w_ of PLA was reduced between 4 and 20%. This reduction and the differences observed are believed to be caused by the dissolution of chloroform-soluble lignin fractions and their inclusion in the analysis.^42^ This conclusion is supported by a larger decrease in *M*_w_ for the films with acetylated lignin due to the increased solubility of acetylated lignin in chloroform. This resulted in the appearance of a low-molecular-weight fraction not present in neat PLA (Figure S2). The thermal transitions of the films were studied by DSC, as shown in Figure 1b. Lignin has been reported to have a nucleating effect and to decrease the surface energy during crystallization, which could explain the increase in crystallinity of around 10% for PLA in all PLA/lignin samples, with the exception of PLA/AL.^22^ PLA/KL-Fract-Acet presented the highest *X*_c_, suggesting that higher compatibility with PLA, due to the decreased molecular weight and acetylation, increased the nucleating effect. The *T*_g_ of PLA and PLA/lignin films, with the exception of PLA/AL, remained constant around 60 °C, similar to what has been observed previously.^24^ The decrease in *T*_g_ and *X*_c_ seen for PLA/AL with respect to neat PLA has been observed in earlier studies.^22^

The surface hydrophobicity, determined by the water contact angle (WCA) of the surface of the films, varied depending on the lignin type. The values determined for PLA are consistent with the literature,^43^ whereas the addition of lignin either decreased (in the case of water-soluble AL), did not alter or slightly increased the WCA (due to surface roughness). These results and further discussion can be found in the SI and Figure S5.

### Hydrolytic Degradation

PLA can be degraded through chemical hydrolysis of the ester bonds, which will lead to formation of water-soluble oligomers and lactic acid as the final degradation product.^44^ This is also typically the first degradation step, enabling subsequent biodegradation. The initial pH of the medium greatly affects the rate of hydrolysis, as a higher hydrolysis rate is found under basic (pH = 10) and acidic (pH = 0.5) conditions.^45^ During hydrolysis, the release of acidic degradation products, e.g., lactic acid, can also influence the pH of the medium. In addition, the presence of many OH groups in lignin can potentially catalyze the ester cleavage (alcoholysis).^46^

#### Molecular Structure of Hydrolyzed Materials: FTIR and 2D HSQC Spectroscopy

To understand the changes in the molecular structure of PLA and lignin during hydrolytic degradation, the functional groups were monitored by FTIR after different hydrolysis times. Despite the prevalence of C–C bonds in lignin, interunit linkages with C–O bonds, such as βO4, and ester bonds introduced during lignin modification, are prone to scission during aging under hydrolytic conditions. If the deacetylation of acetylated lignin takes place, this will lead to the production of acetic acid, which can accelerate the degradation of PLA through acid-catalyzed hydrolysis of ester bonds. Figure 2 shows the FTIR spectra of PLA/AL-Acet as an example (for the spectra of the remaining samples, see Figure S6). All the spectra, before and after hydrolysis, had the characteristic bands of PLA, and all degraded samples also revealed that during hydrolytic degradation, noticeably from day 10, the deacetylation of both aliphatic and phenolic hydroxyls occurred for all acetylated lignins. For all samples before and after hydrolytic degradation, bands corresponding to the asymmetric and symmetric bending of −CH_3_, C=O stretching, −CH_3_ asymmetric deformation, COC stretch, and CH_3_ rocking of PLA were found.^47,48^ Additionally, all the lignin-containing samples showed characteristic lignin bands corresponding to C=C from the aromatic skeleton.^11^ For PLA/AL, this band disappeared after day 1, indicating dissolution of AL. The ratio of the intensities of the band around 921 cm^–1^ (related to the configuration of the α-crystal of PLA) and the band at 957 cm^–1^ ^47^ (related to the amorphous PLA phase) reflects the changes in the degree of crystallinity of the films (Figure S7). All the samples showed a clear increase in this ratio from day 0 to day 30, supporting the increased crystallinity observed by DSC and correlating with the well-known favored degradation of the amorphous phase.^35^ All the acetylated samples showed two peaks at 1017 and 903 cm^–1^, corresponding to OCC and COC of acetyl groups, which decreased during degradation until day 30 (Figure 2c,d).^49,50^ For all the acetylated samples at day 10, a new peak appeared at 1360 cm^–1^, which could be associated with OH of phenolic units originating from deacetylation of aromatic units and possible βO4 cleavage,^51^ see Figure 2b.

**Figure 2:**
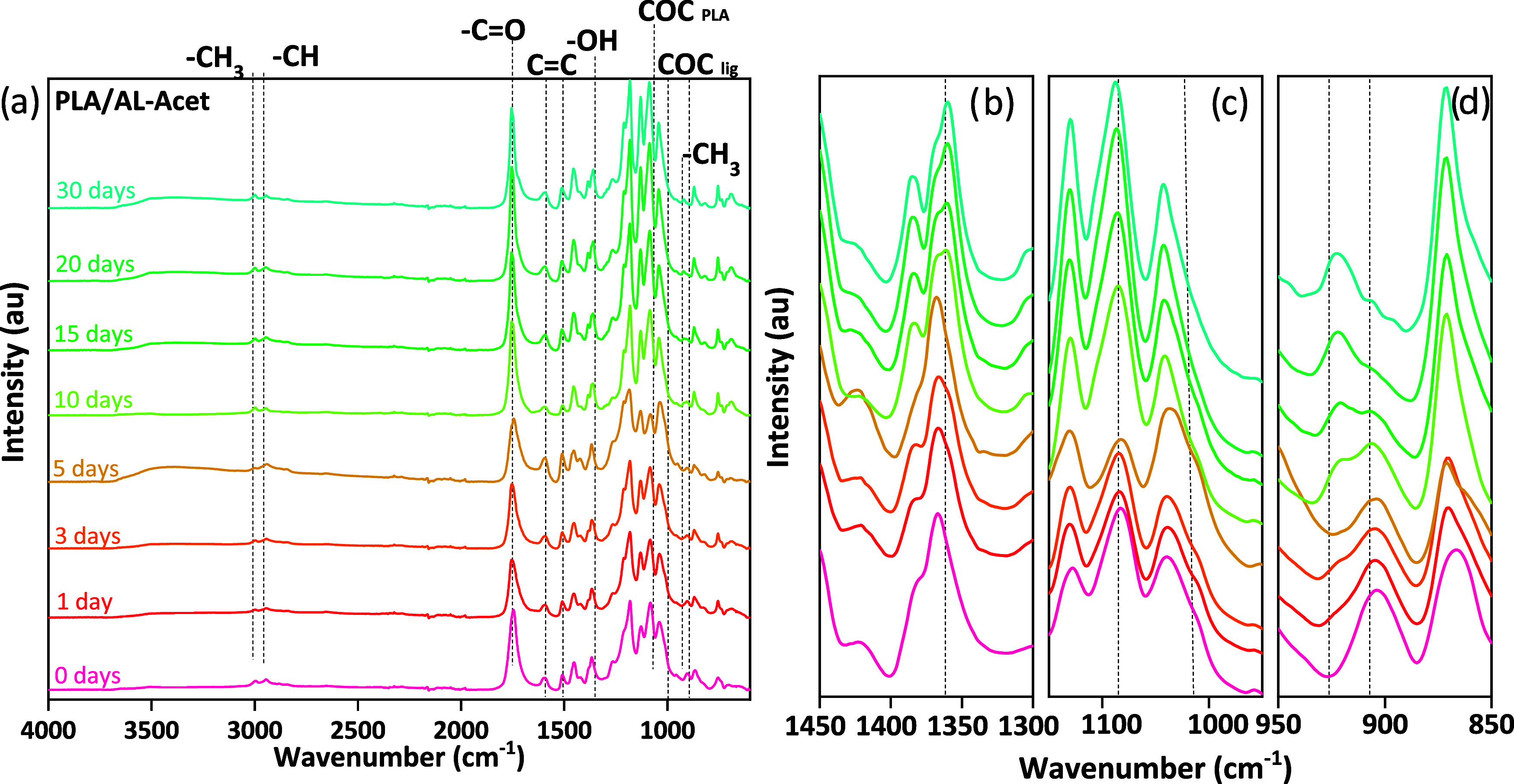
Evolution of functional groups as characterized by FTIR of PLA/AL-Acet: (a) general spectra and (b) absorption band at 1360 cm^–1^, and shifting of bands at (c) 1180 and 1017 cm^–1^ and (d) 920 and 903 cm^–1^.

To assess in more depth the changes in the molecular structure of lignin, analysis and semi-quantification of the most common interunit linkages at day 0 and day 30 were performed by 2D HSQC NMR spectroscopy (Figure 3). AL was not evaluated due to its solubilization in water during the degradation process. The assignment of the different units was done with the help of previous literature and can be found in Table S4.^52^ It is well-known that, depending on, e.g., the extraction process, lignin attains different structures, and the HSQC spectrum here also revealed clear differences in the molecular structures (Figures S8–S12). More specifically, AL-Acet had at least double the amount of βO4 compared to all the other lignins, and therefore, an increased number of hydrolyzable sites were available compared to the other lignins. This increases the potential catalyzing effect of released acetic acid. After hydrolytic degradation for 30 days, partial deacetylation of all acetylated samples, including phenolic and aliphatic hydroxyls, was confirmed by 2D HSQC NMR semi-quantification. In addition, preferential liberation of βO4-rich lignin fractions into the aqueous medium was shown. Semi-quantification of lignin interunit linkages in unaged PLA/KL agreed with previously reported results, such as β-aryl ethers (BO4), resinols (ββ), and phenyl coumaran (β-5).^53^ Semi-quantification of unaged PLA/KL-Fract revealed similarities with PLA/KL; however, a fraction of βO4 groups had been ethoxylated. This correlates with our previous study, where some ethoxylation was shown to take place when ethanol was used as a solvent during microwave-assisted fractionation.^38^ This could contribute to the increased compatibility of βO4-ethoxylated fractions present in KL-Fract with PLA. In addition, the HSQC spectrum showed a new peak around 59,8_C_/4,05_H_, corresponding to ester formation as a result of the condensation reaction between an acid and an alcohol during fractionation^38^ (Figure S11). For all acetylated samples, hydroxyl substitution was followed by the signal at around 20,2_C_/2,25_H_, corresponding to acetyl groups and the shift of the aromatic region C_5_ and C_6_ to C_3_ and C_6_ in the guaiacyl aromatic units,^54^ in addition to the βO4 acetylated peaks. KL-Acet and KL-Fract-Acet had the lowest number of βO4 units, indicating side-reactions during microwave acetylation and fractionation. For both samples, the semi-quantification could not be extended to all major interunit linkages, since the corresponding peaks could not be observed in their NMR spectra. However, the ethoxylation of βO4 was detected in the spectrum of PLA/KL-Fract-Acet.

**Figure 3:**
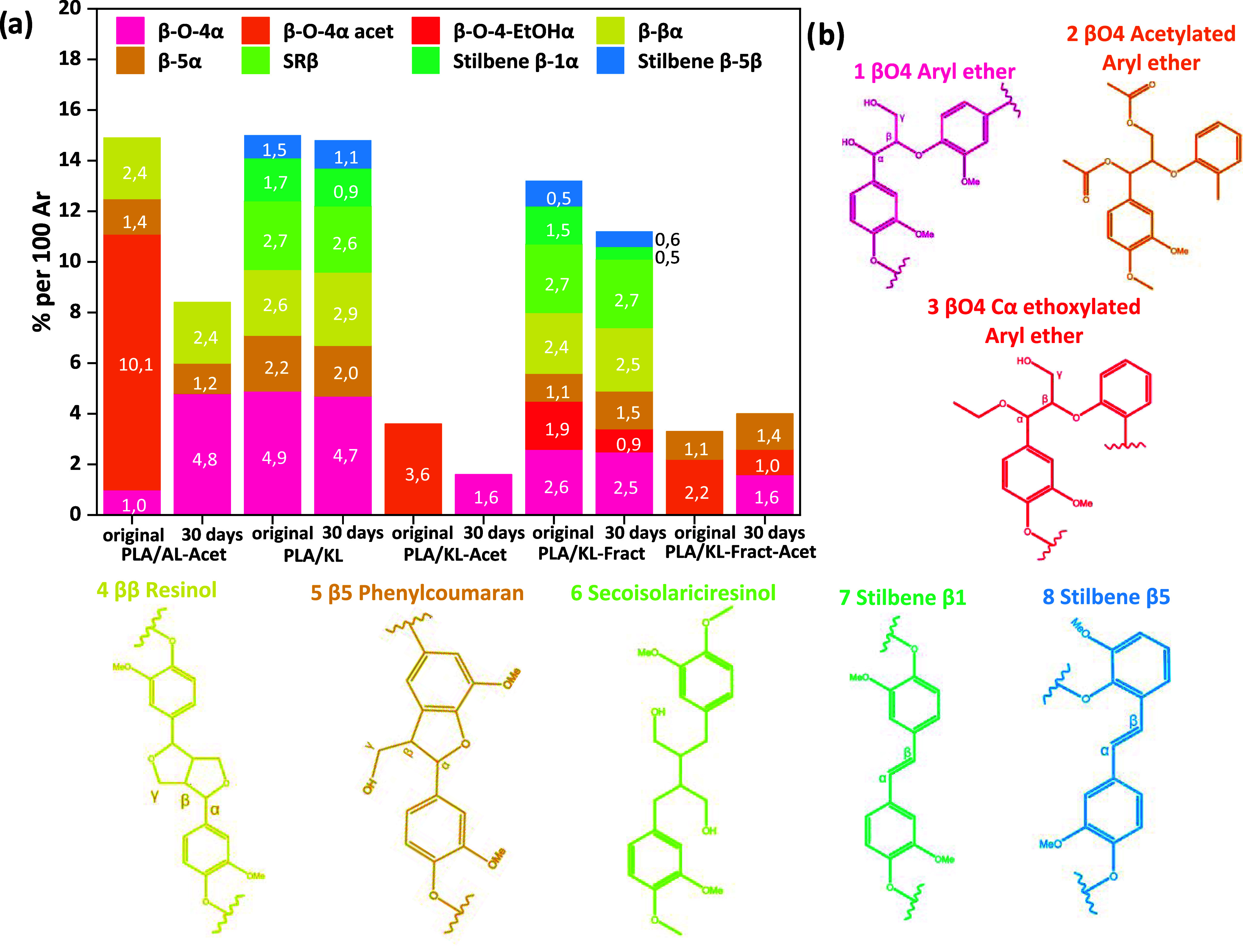
(a) Semi-quantification of basic interunit linkages of lignin in the original and 30-day-aged PLA/lignin samples. (b) Representation of basic interunit linkages in lignin.

After hydrolytic degradation, no significant changes in the interunit linkages of PLA/KL were found, except for a small decrease in stilbene B1 and B5. Similarly, PLA/KL-Fract presented a decrease of stilbene B1 and B5 units, while the amount of βO4 remained constant. However, the amount of ethoxylated βO4 units decreased at day 30 of degradation, indicating the possible liberation of lignin-containing ethoxylated βO4 into the water. In addition, a substantial decrease in the signal intensity of the aromatic ester and ether unit at 61,1_C_ /3,80_H_ was seen, indicating possible scission of ester bonds. After degradation, deacetylation of βO4 units, aryl propanol units, and aromatic units in PLA/KL-Acet was seen, and semi-quantification indicated the loss of lignin fractions with a high content of βO4, as a decreased amount of βO4 units was detected.

For PLA/KL-Fract-Acet, deacetylation reactions were confirmed by decreasing the amount of acetylated βO4. However, the total amount of βO4 units was not altered during the degradation. At day 30 of hydrolytic degradation, PLA/AL-Acet semi-quantification revealed a decrease of acetylated βO4 units and partial deacetylation of aromatic units. Moreover, the total number of βO4 units decreased by approximately 50% with respect to the original materials, again indicating the loss of βO4-rich lignin fractions.

#### Evolution of Molecular Weight

The changes in the molecular weight of PLA due to hydrolytic ester cleavage are indicative of the degree and rate of PLA degradation. Here, we observed an initial acceleration of the degradation rate for PLA films with acetylated and/or fractionated lignin, followed by a slower degradation rate at later stages. This was combined with a considerable increase in the molecular weight of non-fractionated lignins (Figure S15). For KL, the increase in molecular weight is thought to be caused by lignin aggregation after the 30 days of degradation in water; a more detailed explanation can be found in the SI. However, for KL-Acet, and especially AL-Acet, changes in the intramolecular interactions of the lignin during partial deacetylation seem to change the molecular weight distribution, although these changes are thought to be translated to variations in the lignin aggregation and therefore changes in the hydrodynamic volume rather than real changes in the molecular weight. These results support that the acetylated and/or fractionated lignins, particularly the acetylated lignins from which acetic acid was released, catalyzed ester hydrolysis and thereby the molecular weight reduction for PLA. Furthermore, control experiments on PLA degradation with acetic acid embedded in the PLA matrix or present in the surrounding aqueous medium supported the catalytic effect of the released acetic acid on the hydrolytic degradation rate of PLA (see Figure S13 and a more detailed explanation in the SI). For all of the films, the decrease in molecular weight was faster during the first 15 days, after which a slower reduction rate was observed. This is a typical molecular weight evolution pattern during hydrolytic degradation of PLA.^55,56^ The films containing non-acetylated and non-fractionated lignin showed similar patterns of molecular weight decrease (Figure 4a,b). During the analysis of the results, *M*_p_ and *M*_w_ were used instead of *M*_*n*_, due to the presence of chloroform-soluble lignin that affected *M*_*n*_ more than *M*_w_ and *M*_p_, causing a reduction in the calculated *M*_*n*_ value, underestimating the real value. From day 15, the decrease of *M*_p_ and *M*_w_ was consistently larger for the reference PLA films compared to the other samples, indicating a change in the rate and amount of degradation (Table S2). On the other hand, PLA/AL showed the lowest decrease of *M*_w_ and *M*_p_ until day 20. Similarly, PLA/KL showed a slower decrease in molecular weight when compared with neat PLA. All PLA/lignin films with fractionated and/or acetylated lignin showed similar degradation patterns, with a larger decrease of *M*_p_ and *M*_w_ until day 5–10, corresponding to the period when deacetylation was seen with FTIR, compared to the samples with non-acetylated lignin. However, PLA/KL-Fract presented, after day 5, somewhat lower molecular weight decreases compared to all acetylated samples until day 20, due to the absence of acetic acid-catalyzed hydrolysis.

**Figure 4:**
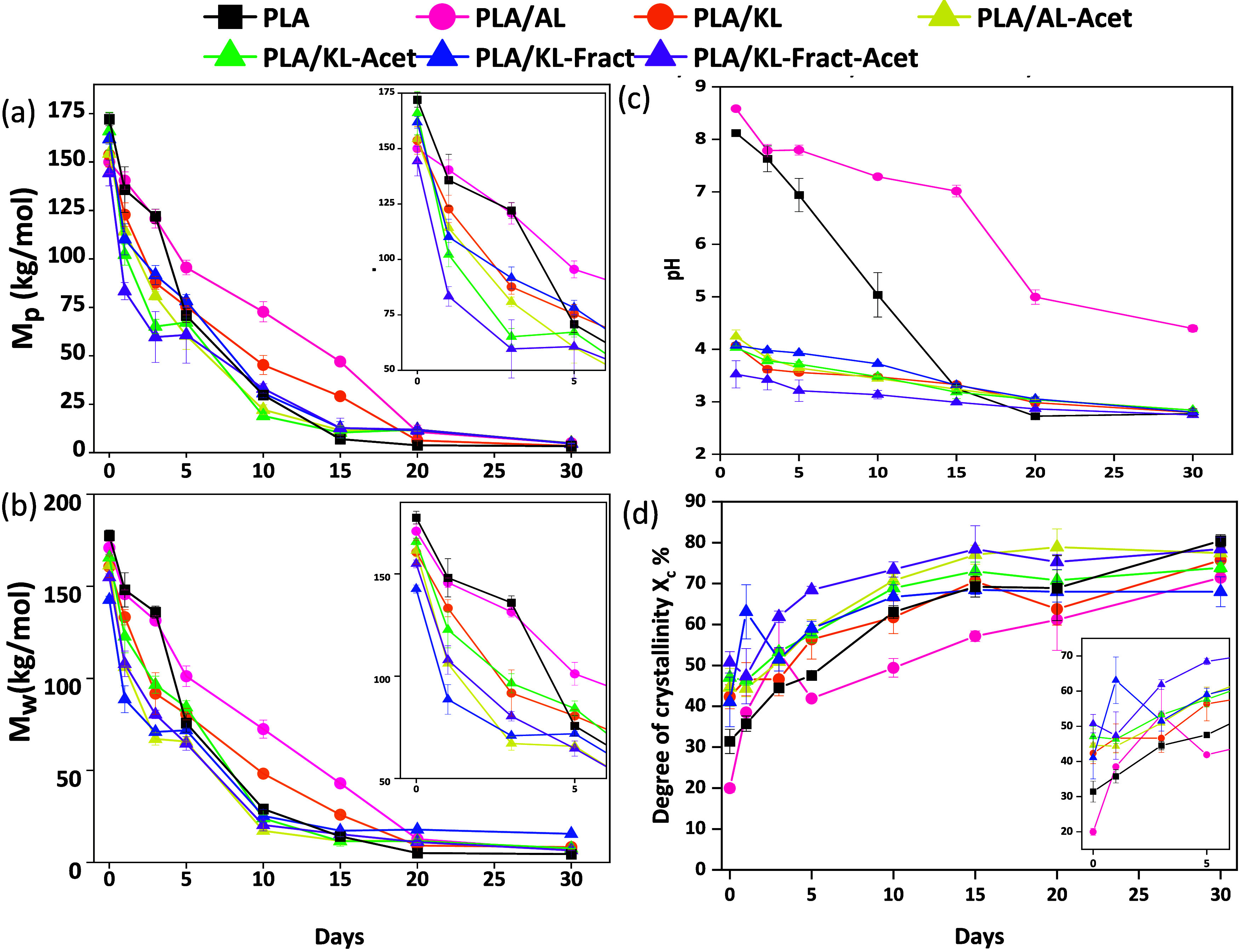
Evolution of (a) *M*_p_ of the chloroform-soluble fraction in PLA and in PLA/lignin samples during hydrolytic degradation obtained with SEC analysis, (b) *M*_w_, (c) pH of aqueous medium, and (d) degree of crystallinity (*X*_c_).

#### pH Measurements

During hydrolytic degradation of PLA, the cleavage of ester bonds will promote the liberation of lactic acid and soluble acidic oligomers in the medium,^57^ which will decrease the pH of the water and accelerate the degradation through acid-catalyzed ester hydrolysis. Monitoring the pH change revealed how the aging medium of all PLA/lignin samples, except PLA/AL, illustrated a pH decrease already at day 1, compared with neat PLA (Figure 4c). On the other hand, AL that was solubilized in water, from PLA/AL, seemed to display a buffering effect when acidic products were released to the medium, which can explain the decreased rate of degradation in comparison to the rest of the samples. In addition, the loss of lignin from the PLA matrix could translate to the loss of OH groups, catalyzing ester cleavage of the polymer backbone. To ensure a correct comparison, the pH of pure distilled water and the pH after adding KL, KL-Acet, and AL was also recorded. For pure distilled water, the pH was found to be around 8, while it became more basic for AL with a pH of 8.5. KL and KL-Acet alone registered similar pH to those seen for PLA/KL and PLA/KL-Acet at day 1. A pH decrease to around 3 during degradation of the neat PLA film agrees with previous studies.^47^ To assess the effect of the dissolved AL in the medium, 1 g of AL was dissolved in 40 mL of distilled water, leading to a pH of 10.1, and 3 × 50 μL of lactic acid was added progressively while measuring the pH. After addition of 150 μL of lactic acid, the pH was reduced to 9.1. In comparison, after adding only 50 μL of lactic acid to 40 mL of pure distilled water with an original pH of around 8, the pH was reduced to 3.1. This confirmed the buffering effect that AL could have during the hydrolysis of PLA, which could decrease the hydrolytic degradation rate. The display of a buffer effect in lignin has been already reported, and related to the different hydroxyl groups present in lignin (mainly aromatic and carboxylic).^58,59^ In detail, water-soluble lignin was reported to maintain a pH close to 8.9 due to the partial deprotonation of phenolic and carboxylic groups, which could resist changes in the pH of the solution and therefore act as a buffer.^58^ The initial pH of PLA/KL was much lower than the pH for neat PLA, and it further decreased as a function of the degradation time. Acetylated and/or fractionated samples followed a trend similar to that of PLA/KL; however, PLA/KL-Fract-Acet showed the lowest values of pH throughout the degradation period, supporting the larger amount of acetic acid released.

#### Thermal Properties of Hydrolyzed Films

To understand the effect of the different lignins on the thermal properties of PLA/lignin films during hydrolytic degradation, all samples were analyzed by TGA and DSC. During hydrolysis, the degree of crystallinity increased for all of the samples. A faster increase in the degree of crystallinity was seen for all acetylated samples and to some extent also for the fractionated samples (Figure 4d). This was combined with an initial faster decrease in *T*_g_ (Figure S16). This agrees with the initial hydrolysis-accelerating effect and faster molecular weight decrease. However, we believe that the increased interface between PLA and lignin aggregates in the acetylated and/or fractionated samples, due to improved compatibility, facilitated the crystallization process, which would explain why *X*_c_ for PLA/KL-Fract-Acet was higher at almost all stages compared with the rest of the films. During degradation, water penetrates the more accessible amorphous phase of PLA, facilitating chain scission in those areas.^45^ Therefore, upon degradation, amorphous areas are more likely to be degraded, which causes an increase in *X*_c_. The evolution of the crystallinity also indicates that after 30 days, the amorphous region had been preferentially degraded in all samples. For PLA/AL, despite the solubilization of AL from day 1, the increase in *X*_c_ was the slowest compared to the rest of the samples due to the observed buffering effect of AL. Neat PLA and PLA/KL presented a similar increase in *X*_c_. All acetylated and/or fractionated PLA/lignin films showed similar trends of evolution of *X*_c_, increasing from day 0 to around day 15 and reaching a plateau at day 30.

The decrease in molecular weight due to hydrolysis decreases the *T*_g_ due to a higher chain mobility and a larger amount of chain ends, increasing the free volume. In addition, there can be a plasticizing effect from the uptake of water molecules and the formation of PLA oligomers.^60^ Altogether, the *T*_g_ decrease followed a similar pattern as was seen for the decrease of the molecular weight of the PLA fraction. Compared to neat PLA, the final *T*_g_ of all PLA/lignin samples was somewhat higher.

The decrease in thermal stability upon hydrolysis of PLA was in line with the scission of ester bonds in the amorphous region.^60^ However, based on *T*_5%_ and *T*_max_ values, the thermal stability of the PLA/lignin films after hydrolytic aging was higher compared to neat PLA films, with the exception of PLA/AL (Figure S17).

#### Evolution of Film Morphology

The top surface (in contact with air during solvent casting) and cross section (Figure 5) were imaged by FE-SEM. In all samples, lignin aggregation was observed, creating an interface area between the PLA matrix and lignin, and acetylation of lignin was expected to increase the compatibility with the PLA matrix. The average diameter of the lignin aggregates (Table S5 and Figure S19) revealed how a higher and broader molecular weight dispersity and the presence of OH groups all lead to bigger and more heterogeneous lignin aggregates (Table S5 and Figure S19). While PLA/AL (Figure 5b) and PLA/KL (Figure 5c,d) presented aggregates that were either too big or too heterogeneous to be evaluated, PLA/AL-Acet and PLA/KL-Acet (Figure 5g,f, respectively) portrayed aggregates with diameters of 7.4 ± 4.7 and 7.9 ± 5.1 μm, respectively. This supported the presence of more uniform distribution of small round-shaped lignin aggregates, which, in turn, increased the interface area between the two components.^25,61^ In addition, the particle size distribution of the different lignin materials in DCM was measured to understand how the different modifications and molecular weight distributions altered the solvent–lignin interactions (Table S6 and more detailed information in the SI). For PLA/KL-Fract-Acet (Figure 5h,i), with a lower and narrower dispersity and a reduced number of OH groups, the average aggregate size was 4.7 ± 1.3 μm. This is a significant reduction that will increase the surface area. The surface of PLA/KL-Fract presented small round-shaped aggregates, of 2.1 ± 0.5 μm size, believed to originate from higher compatibility with PLA, possibly facilitated by the decrease in the molecular weight and ethoxylation seen with 2D HSQC (Figure 5e). In addition, increased rugosity of the surface was seen for PLA/KL, PLA/KL-Fract, and PLA/KL-Fract-Acet with respect to the rest of the samples, which presented flat surfaces. A more detailed description of the morphology of the films is given in the SI.

**Figure 5:**
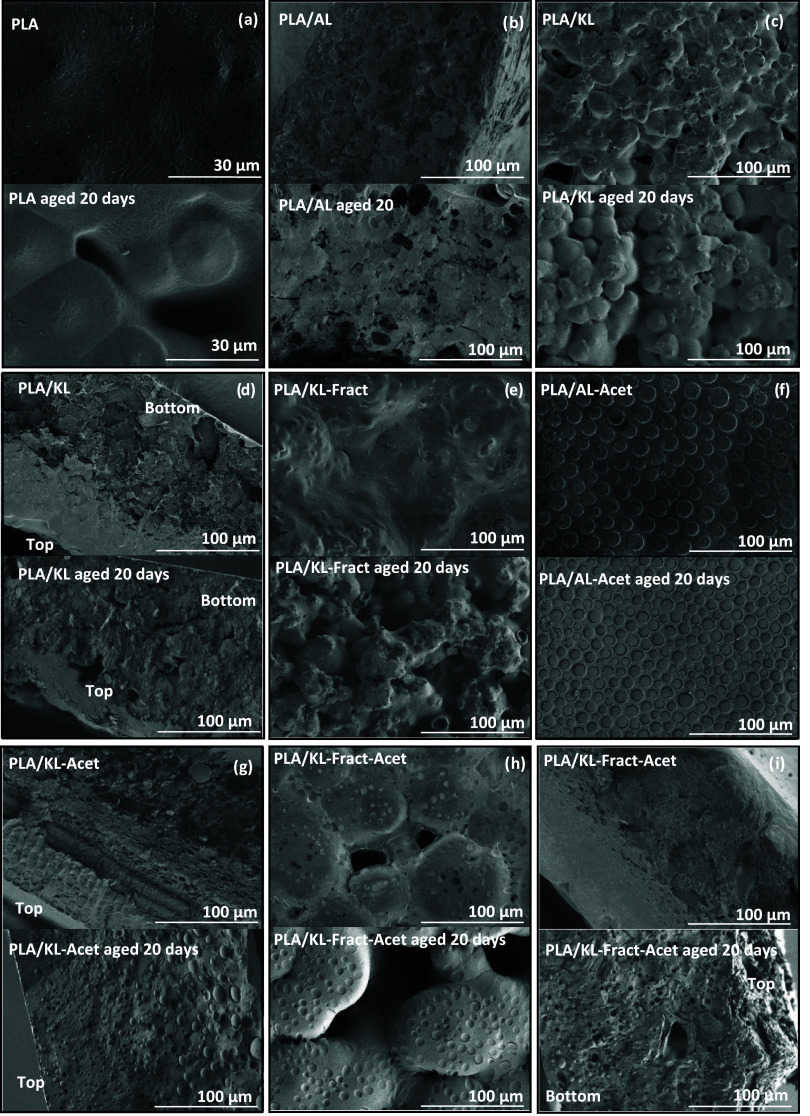
(a–i) SEM images showing the top surface or cross-sectional morphology of the original and hydrolytically aged PLA and PLA/lignin films at day 20.

Changes in surface and cross-sectional morphologies on the PLA and PLA/lignin films during hydrolytic degradation were monitored after different time periods. In addition, the liberation of lignin from the degradation medium of PLA/KL-Fract was demonstrated. In aged neat PLA films, the surface after 20 days presented a loss of the amorphous phase with a subsequent increase of crystallinity. Changes in the cross section of PLA/AL also indicated an increase in crystallinity (Figure 5b). For PLA/KL, surface changes followed a similar trend to that of neat PLA (Figure 5d). Similarly, the PLA/KL-Fract cross section showed increased depletion of lignin, forming surface cavities on the film thickness and indicating lignin liberation into the aqueous medium during degradation (Figure 5e). PLA/AL-Acet and PLA/KL-Acet changed similarly throughout the degradation. The evenly distributed round lignin aggregates on the surface seemed to hinder erosion of the surface material. Images of the aged surface showed how the interface of the lignin aggregates with the PLA matrix seemed to be partially broken (Figure 5f). An explanation could be the preferential absorption of water in the top surface, due to the large PLA/lignin interface.^62^ Lignin deacetylation, leading to the loss of compatibility between PLA and lignin, could also have resulted in the loss of adhesion at the interface. The aged surface of PLA/KL-Fract-Acet indicated further loss of the material and increased the presence of spherulites. Similar to the acetylated samples, the surface showed a clear loss of adhesion between lignin aggregates and PLA. It is again plausible that deacetylation of lignin occurred at the interface due to the preferential water absorption in those areas.

#### Water Absorption and Weight Loss

During hydrolytic degradation of PLA, first, water is absorbed through the surface, followed by weight loss due to hydrolysis predominantly in the amorphous phase, after a while, causing solubilization of degraded products. Water absorption was followed as a function of time for all films (Figure S20), and all PLA/lignin films presented higher water absorption compared to that of the neat PLA film. The effect of surface morphology, lignin molecular weight, and acetylation all played a role in the water absorption process, where higher roughness was the factor with the largest influence. PLA/AL presented the highest water absorption at all times, followed by PLA/KL-Fract-Acet and PLA/KL-Fract; a more detailed analysis is given in the SI.

Water absorption trends did not correlate with the weight loss of the films, opposite to what has been observed for PLA blends^63^ (Figure 6), and clear differences in the hydrolytic degradation process for non-modified and acetylated and/or fractionated films were seen. Since lignin degradation is hindered by its recalcitrant nature and less hydrolysis-sensitive bonds compared to PLA, weight loss is assumed to mainly be caused by degradation of PLA. PLA, PLA/AL, and PLA/KL all showed similar patterns of weight loss throughout the 30 days, with a plateau reached at day 20. Here, it is important to point out that the weight loss of PLA/AL combines the contribution caused by degradation and the dissolution of AL. To evaluate the amount of AL lost due to dissolution, 3 PLA/AL samples were placed in 20 mL of distilled water at 60 °C for 30 min, and the average mass loss was calculated to be 27.1 ± 2.3%. The plateau could be connected to a decrease in the rate of degradation due to the consumption of most of the more accessible amorphous phase. The fractionated and/or acetylated PLA/lignin films all presented similar linear weight loss patterns. The larger weight loss at day 30 for the fractionated and/or acetylated lignin films, especially for the acetylated PLA/lignin films, further supports the ester hydrolysis-catalyzing effect of acetic acid released by deacetylation. The linear weight loss trend for these samples could also indicate that degradation of the amorphous phase had not reached the saturation point or that lignin was liberated into the water along with the formation of soluble degradation products from PLA, which agrees with the results seen with FE-SEM and 2D NMR HSQC. The PLA/AL-Acet films exhibited the highest weight loss, likely a consequence of a gradual deacetylation of AL, causing the release of deacetylated lignin fractions into the aqueous medium.

**Figure 6:**
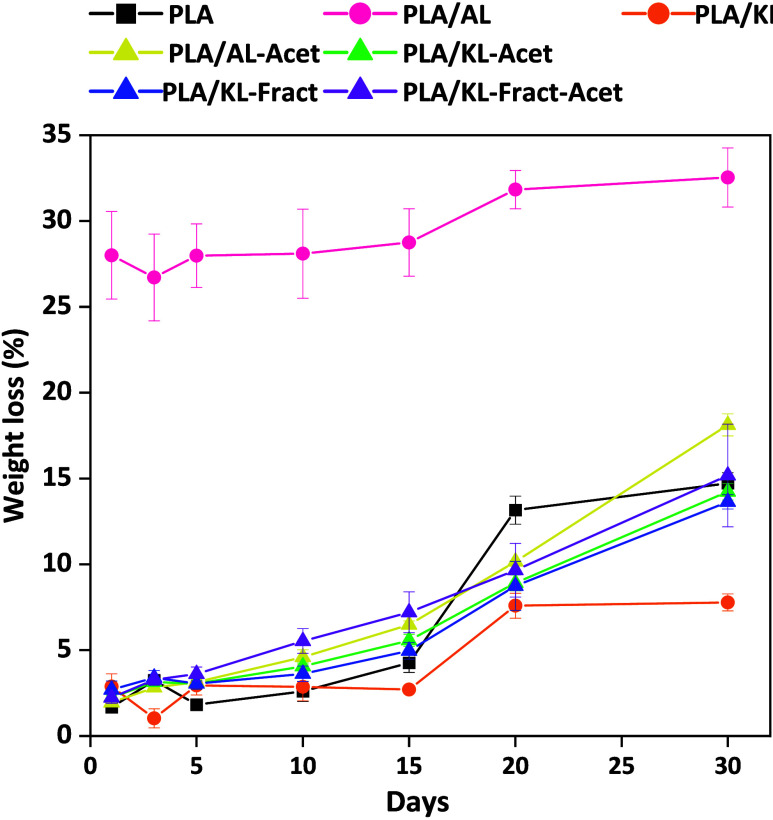
Evolution of weight loss during hydrolytic degradation of PLA and PLA/lignin films.

#### Interplay between the Degradation Mechanism and Lignin Type

In this work, due to the sample geometry, bulk degradation was considered to occur for all samples, and the primary mechanism of ester scission occurring was proposed to be main chain ester cleavage, since for all the samples, there was a considerable reduction of molecular weight starting from the first days. When PLA is placed in water, there are usually four identified phases that occur: (1) water is absorbed, (2) the amorphous phase is preferentially degraded, (3) ester bond cleavage causes a decrease of molecular weight and formation of soluble oligomers, and, last, (4) the remaining ordered crystallites are subjected to hydrolysis.^47^ Until day 30, the first stages of hydrolytic degradation of PLA were identified, where water is absorbed, followed by weight loss and an increase in *X*_c_. It was clearly shown how the incorporation of different lignin types affects the degradation mechanism of PLA in different ways. Despite the initial lower pH of KL and acetylated and/or fractionated lignin with respect to neat PLA, there were other factors affecting the degradation process, such as the initial physicochemical properties and morphology of the film, water absorption, and acid-catalyzed hydrolysis. We believe that for acetylated samples, the presence of a larger PLA/lignin interface on the surface of the films and the increased surface roughness, in the case of PLA/KL-Fract-Acet, confirmed by FE-SEM, increased the water absorption in those areas. This further facilitated the hydrolysis of the ester groups in the acetylated samples, as confirmed by FTIR and 2D HSQC NMR. The liberation of acetic acid increased the acidity at the PLA/lignin film and catalyzed the hydrolysis rate at early stages. The presence of a large PLA/lignin interface and the increased miscibility of the phases for acetylated samples accelerated the degradation and promoted a fast increase of *X*_c_. This effect was accentuated for PLA/KL-Fract-Acet due to the increased interface area. While for PLA/KL-Fract, there was no acid-catalyzing effect, the combination of the increased interface between the lignin aggregates and PLA, the increased water absorption with respect to the non-fractionated samples, and the possible increased alcoholysis effect of the OH groups with respect to KL could account for the accelerated degradation of the PLA. In addition, the hydrolysis of ester groups in lignin and the liberation of lignin fractions could explain the increased weight loss seen for fractionated and acetylated lignins. On the other hand, PLA/KL showed a slower degradation with respect to PLA, probably due to the combination of an increased degree of crystallinity of the original film with respect to neat PLA and the smaller interface of the lignin aggregates with PLA. For PLA/AL, despite the lignin solubilization, the buffering effect of AL hindered the acid-catalyzed hydrolytic cleavage, reducing the rate of ester hydrolysis.

### Degradation under Simulated Industrial Composting Conditions

The degradation rate of PLA at low temperatures under, e.g., soil burial is relatively low; therefore, industrial composting conditions were selected.^64^ Here, we especially wanted to confirm that degradation reactions, such as deacetylation confirmed under hydrolytic conditions, also take place under composting conditions. Under these conditions, PLA is initially expected to experience hydrolytic degradation due to the inability of most microorganisms to digest long polymer chains.^64^ The composting environment is a very complex system; however, a preliminary test to evaluate the degradation process under such conditions provides insights into the degradation process and potential compostability of the materials. In future work, CO_2_ evolution should be followed to fully evaluate and confirm the biodegradation process and compostability.

#### Molecular Structure of PLA and PLA/Lignin Films

Changes in the composition and functional groups of PLA and PLA/lignin films were investigated by FTIR at days 63 and 105 of simulated industrial composting (Figure S21). Analysis of the changes in the FTIR spectrum revealed similar changes to those seen after hydrolytic degradation (Figures 7 and S21). All aged PLA and PLA/lignin films presented an additional band around 1600 cm^–1^, thought to be caused by residual compost and water absorption. Deacetylation of all acetylated samples was confirmed on day 63. PLA/AL-Acet and PLA/KL-Acet presented a new band around 1361 cm^–1^, associated with OH of phenolic units (Figure 7a). Moreover, the shoulder at 1017 cm^–1^ disappeared by day 63 (Figure 7b). The three acetylated films showed a decrease in the band intensity at 903 cm^–1^, corresponding to the COC of acetyl groups (Figure 7c). The deacetylation reactions could be catalyzed by hydrolyses present in the compost.

**Figure 7:**
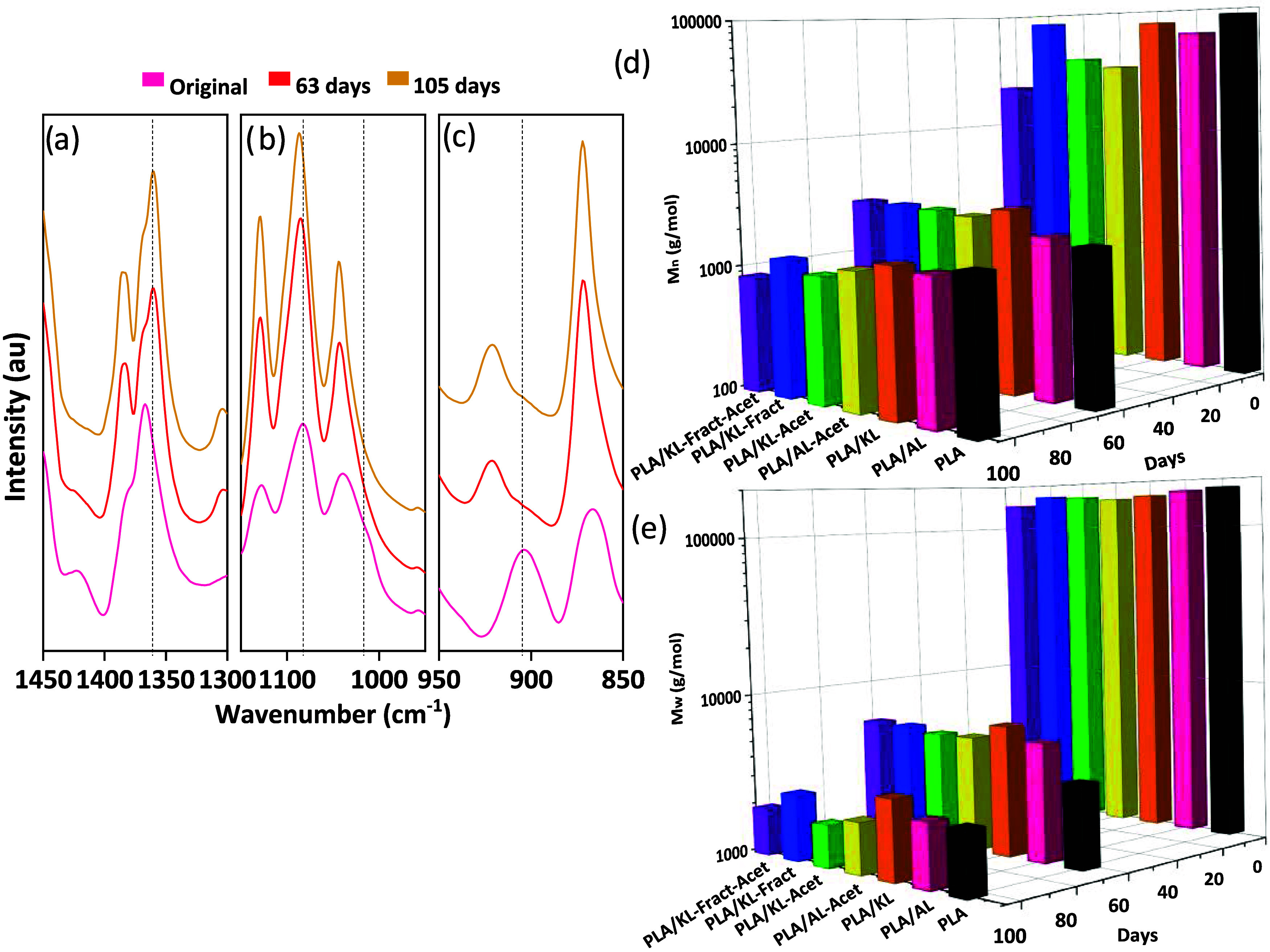
Evolution of the functional groups monitored by FTIR spectroscopy during simulated composting of PLA/AL-Acet. Shifting of the absorption bands (a) at 1360 cm^–1^, (b) at 1180 and 1017 cm^–1^, and (c) at 920 and 903 cm^–1^. Evolution of molecular weight parameters (d) *M_n_* and (e) *M*_w_.

#### Molecular Weight of Aged PLA and PLA/Lignin Materials

The changes in the molecular weight of PLA and lignin fractions during simulated industrial composting were investigated by SEC following the same procedure as that after hydrolytic degradation. Evolution of the molecular weight distribution of PLA and lignin fractions is shown in Figure S22a,b, respectively, and evolution of *M*_*n*_ and *M*_w_ can be found in Figure 7d,e. Similar to the results seen for hydrolytic degradation, an increase in the molecular weight of lignin was observed, especially in the cases of PLA/AL-Acet and PLA/KL-Acet. Samples without or with a small amount of lignin during the hydrolysis process (PLA and PLA/AL) showed a higher decrease in *M_n_*, compared to the other samples at day 63, which could be connected to the lower degree of crystallinity. However, by day 105, the acetylated samples showed a higher reduction of molecular weight. All samples had a relatively similar pattern of a molecular weight decrease throughout the degradation period. It has been described in the literature how during composting of PLA, first the molecular weight needs to be substantially reduced to 15,000–40,000 by hydrolytic degradation before the microorganisms can digest the remaining polymer chains.^64^ From the obtained results, we could see how all of the samples had reached such values by day 63. The deacetylation reactions confirmed for modified samples did not seem to negatively affect the reduction of molecular weight due to liberation of acetic acid in the medium. On the contrary, a higher decrease in molecular weight was seen in comparison to non-modified samples.

## Conclusions

The degradation processes of both PLA and lignin demonstrated differences depending on lignin type. Changes in the lignin molecular structure due to acetylation or fractionation greatly affected the morphology and distribution of the lignin aggregates in the PLA matrix. As expected, a decrease in OH groups in lignin due to acetylation and ethoxylation for fractionated samples increased the compatibility with PLA, where KL-Fract-Acet showed enhanced compatibility with PLA compared to KL-Acet. Higher compatibility between PLA and acetylated lignin led to a larger interface between the two components, which altered the film properties by promoting higher degrees of crystallinity and water absorption during hydrolytic degradation. During degradation, deacetylation of lignin was confirmed to occur at early degradation stages, with the subsequent increase in PLA ester cleavage due to acetic acid-catalyzed hydrolysis, causing a more rapid initial decrease of PLA molecular weight. On the other hand, the presence of non-modified Kraft and alkaline lignin initially slowed down the degradation of PLA. The lignin deacetylation reaction was also shown to occur during composting conditions. Moreover, the deacetylation of lignin led to the release of OH groups, which in turn could further benefit the degradability of the material. This work shows the importance of performing an integral evaluation of the degradation processes of all of the material components, from the changes in the molecular structure to the visualization of the material morphology. By understanding the influence of different lignin molecular and macromolecular architectures, the physicochemical properties and morphology of the final material can be tailored and the degradation process tuned depending on the desired application and end-of-life environment.

## Supplementary Material



## Notes

^31^P NMR spectra of different lignin materials; molecular weight distributions of original PLA and PLA/lignin films and PLA fraction and lignin fraction of aged samples; thermogravimetric analysis of original PLA and PLA/lignin films and aged samples, WCA of PLA and PLA/lignin films; FTIR spectrum of hydrolytically degraded PLA/AL, PLA/KL, PLA/KL-Acet, and PLA/KL-Fract and aged samples; intensity ratio of peak 921 and 957 cm^–1^ during hydrolytic degradation of PLA and PLA/lignin films; 2D NMR HSQC of original and hydrolytically aged PLA/lignin films; glass transition temperature of hydrolytically aged PLA and PLA/lignin films; FE-SEM images of PLA, PLA/AL, PLA/KL-Fract, PLA/KL-Acet, PLA/AL-Acet, and PLA/KL-Fract; and water absorption evolution of PLA and PLA/lignin films (PDF)
