# Supplementary material for: Interplay between the Molecular Structure of Lignin and the Degradation Process of PLA/Lignin Materials
Source: Biomacromolecules. 2026 Apr 14;27(5):3147–60. doi: 10.1021/acs.biomac.5c02467 (PMC13169371; doi:10.1021/acs.biomac.5c02467)
Supplement: Supplementary file 1 [file bm5c02467_si_001.pdf]

Supporting Information

Interplay between molecular structure of lignin and degradation process of PLA/lignin materials

Paula Pou I Rodríguez[1,2], *Karin Odelius*[1,2], *Minna Hakkarainen*[1,2] *

[1] Wallenberg Wood Science Center (WWSC), KTH Royal Institute of Technology, Teknikringen 56, 100 44 Stockholm, Sweden

[2]Department of Fiber and Polymer Technology, KTH Royal Institute of Technology, Teknikringen 58, 100 44 Stockholm, Sweden

*Corresponding author

This supporting information contains 23 Figures and 6 Tables on 30 pages.

List of Figures

Figure S1. ^{31}P-NMR spectra of different lignin materials. 3
Figure S2. Molecular weight distributions of original PLA and PLA/lignin films obtained with SEC Chloroform. 6
Figure S3.Thermogravimetric analysis of PLA and the different PLA/lignin films 6
Figure S4. Thermal stability presented by $T_{5\%}$ and T_{max} of original PLA and PLA/lignin films as determined by TGA. 7
Figure S5. WCA for original PLA and PLA/lignin films 8
Figure S6. Evolution FTIR spectrum during hydrolytic degradation for a) PLA/AL, b) PLA/KL, c) PLA/KL-Acet, d) PLA/KL-Fract and e) PLA/KL-Fract-Acet........ 9
Figure S7. Evolution of the intensity ratio of peak 921 cm^{-1} and 957 cm^{-1} during hydrolytic degradation of PLA and PLA/lignin degraded samples. 10
Figure S8. 2D-NMR HSQC spectrum of a) original PLA/KL and b) aged PLA/KL at day 30 under hydrolytic degradation. 12
Figure S9. 2D-NMR HSQC spectrum of a) original PLA/AL_Acet and b) aged PLA/AL_Acet at day 30 under hydrolytic degradation........ 13
Figure S10. 2D-NMR HSQC spectrum of a) original PLA/KL_Acet and b) aged PLA/KL_Acet at day 30 under hydrolytic degradation. 14
Figure S11. 2D-NMR HSQC spectrum of a) original PLA/KL_Fract and b) aged PLA/KL_Fract at day 30 under hydrolytic degradation. 15
Figure S12. 2D-NMR HSQC spectrum of a) original PLA/KL_Fract_Acet and b) aged PLA/KL_Fract_Acet at day 30 under hydrolytic degradation. 16
Figure S13: Evolution of molecular weight of PLA/neutral, PLA/acid and PLA/+acetic.a samples during hydrolytic degradation with SEC Chloroform: a) M_n and b) M_w. 17
Figure S14. Evolution of molecular weight distributions of PLA and PLA/lignin samples during hydrolytic degradation obtained with SEC analysis using the chloroform soluble fraction........ 17
Figure S15: Evolution of molecular weight of the chloroform soluble fraction of PLA and PLA/lignin samples during hydrolytic degradation: a) M_p and b) M_w........ 17
Figure S16. a) Evolution of molecular weight distributions of DMSO/ LiBr soluble fractions in PLA/lignin films during hydrolytic degradation with SEC and b) Molecular weight vs elution time for all original PLA/lignin films (except PLA/AL) and hydrolytically aged films at day 18
Figure S17. Evolution of T_g during hydrolytic degradation of all PLA and PLA/lignin films. 20
Figure S18. Thermal parameters, $T_{5\%}$ and T_{max}, of original and aged PLA and PLA/lignin films under hydrolytic degradation at day 30. 21
Figure S19. PLA and PLA/lignin surface and cross-section morphology for original and aged materials imaged by FE-SEM: a) PLA, b) PLA/AL, c) PLA/KL-Fract, d) PLA/KL-Acet, e) PLA/AL-Acet and f) PLA/KL-Fract bottom surface........ 23
Figure S20: Distribution of lignin aggregates diameter in original and aged after 20 days PLA/lignin films. 24
Figure S21. Evolution of water absorption during hydrolytic degradation PLA and PLA/lignin films. 26
Figure S22. Evolution of functional groups of original and aged PLA and PLA/lignin films during simulated industrial composting characterized with FTIR. 26
Figure S23. Evolution of molecular weight distributions during simulated composting of a) chloroform soluble fraction of PLA and PLA/lignin films and b) DMSO/LiBr soluble fractions of PLA/lignin films........ 29

List of tables

Table S1: Assignments of C/H signals of lignin units in all original lignin materials, detected by 2D HSQC spectroscopy in DMSO-d_6 4
Table S2. Average molecular weights, M_n, M_w and M_p of PLA and chloroform soluble fractions of original and 30 days hydrolytically aged PLA and PLA/lignin films........ 6
Table S3. Thermal stability as determined by $T_{5\%}$ and T_{max} of original lignin materials 7
Table S4. Assignments of C/H signals of lignin units in all original and aged at 30 days PLA/lignin films, detected by 2D HSQC spectroscopy in DMSO-d_6........ 10
Table S5: Average diameter of lignin aggregates in original and aged after 20 days PLA/lignin samples........ 25
Table S6: Particle size distribution parameters of different lignin materials in DCM at a concentration of 0.3 mg/mL. 25

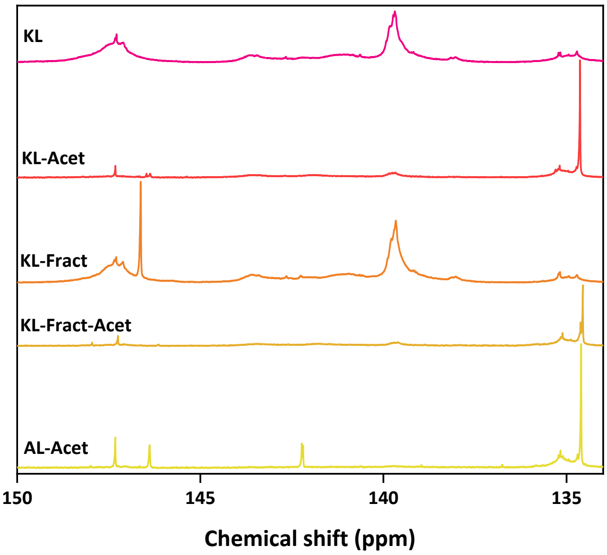

Figure S1. ^{31}P-NMR spectra of different lignin materials.

Quantitative analysis of hydroxyl group content for the different lignin materials was done via ^{31}P-NMR spectroscopy, following a previously reported protocol.[1] Each lignin material (around 30 mg) was dissolved in 0.5 mL of chloroform-*d* and pyridine (1:1.6 v/v). The internal standard solution was added (0.1 mL), which was prepared with the relaxation agent $Cr(acac)_3$ (5.0 mg/mL) and the internal standard NHND (18 mg/ml) in the chloroform-d/pyridine solvent system. After stirring overnight, the TMDP (0.1 mL) was added. The spectrum was recorded on a Bruker Avance III HD 400 MHz spectrometer (Brujer Corporation, U.S.A.) with 1024 scans and 2 s of relaxation delay. MestReNova software was used to processes the data,

including multipoint baseline correction and manual phase correction. The reference single was placed at 132.2 ppm, result of the product of TMDP and water. Quantification of hydroxyl group was calculated by integration of the aliphatic OH (145.4-150.0 ppm), phenolic OH (137.6-144.0 ppm) and carboxylic acid OH (133.6-136.0 ppm) regions and the internal standard (151.5-152.1 ppm). The concentration of OH group (mmol OH/ g lignin) was calculated suing ***equation S1***, where R is the integration ratio between each OH region and the internal standard. The degree of substitution (DS) was then calculated suing ***equation S2***, where $C(OH)_i$ (mmol/g) and $C(OH)_f$ (mmol/g) are the initial and final concentration of OH groups in the lignin samples, respectively, and Δm_{max} is the weight increase (g) per gram of lignin at 100% conversion.

Equation S1 $$mmol\,OH/g\,lignin = \frac{R * NHND\ in\ NMR\ samples\ (mmol)}{lignin\ (g)}$$

Equation S2 $$DS = \frac{C(OH)_i - C(OH)_f}{C(OH)_i + C(OH)_f * \Delta m_{max}}$$

Table S1: Assignments of C/H signals of lignin units in all original lignin materials, detected by 2D HSQC spectroscopy in DMSO-d_6

	KL	KL-Fract	KL-Acet	KL-Fract-Acet	AL	AL-Acet
**O-acetylated**			19,9-20,2/1,9-2,3	19,6-20,2/1,9-2,3		19,9-20,5/1,9-2,1
**CαHα in dihydrocinnamyl alcohol**					31,5/2,5	31,2/2,6
**CβHβ in dihydrocinnamyl alcohol acetylated**			29,5/1,9	29,5/1,9		29,5/1,9
**CαHα in secoisolariciresinol**	33,5/2,5	33,2/2,5			33,2/2,5	
**CαHα in dihydrocinnamyl alcohol acetylated**			33,9/2,8	33,5/2,8		33,9/2,8
**CβHβ in dihydrocinnamyl alcohol**	34,5/1,7	34,5/1,7	35,5/1,7	35,5/1,7	34,2/1,7	
**CβHβ in secoisolariciresinol**	42,2/1,9	42,2/1,9			42,2/1,8	
**CγHγ in OMe in cinnamaldehyde end groups (methoxy instead of H)**	51,1/3,8	51,1/3,8				
**C/H in OMe**	55,5/3,8	55,5/3,8	55,1/3,8	55,5/3,7	55,5/3,7	55,1/3,8
**CβHβ in resinol**	54,1/3,0	53,5/3,1			53,5/3,1	53,8/3,1
**CH_2 in ester from condensation of acid and alcohol from lignin or solvent**		59,8/4,1			60,1/3,4	59,4/3,4
**CγHγ in β-O-4**	60,1/3,4	60,1/3,4-3,6			61,4/4,1	
**CγHγ Phenyl coumaran**	62,8/3,9					62,8/4,0
**CγHγ dihydrocinnamyl alcohol**	61,4/4,1					
**Ethyl ethers from o-alkylation of phenolic OH**		61,1/3,8				

**Ethyl ethers from substitution of the α carbon in β-O-4**		63,4/3,3		63,8/3,4		
**Cγ-Hγ in γ-acylated β-O-4**			62,8/4,0	62,4/4,0		
**C_6/H_6 β-D-mannopyranoside**					68,1/3,7	
**CγHγ in resinol**	70,4/3,8-4,2	70,7/3,8-4,4				70,4/3,8-4,2
**CαHα in β-O-4**	71,1/4,8	71,1/4,8	71,1/4,8	70,7/4,8	70,7/4,8	
**C_2/H_2 in β -D xylopyranoside**	72,7/3,1					
**C_3/H_3 in β -D-xylopyranoside**	73,7/3,3					
**C_4/H_4 in β -D xylopyranoside**	75,1/3,6					
**CαHα in aryl hydroxy acetic acid**	73,7/4,4	73,7/4,4				
**CαHα in γ-acylated β-O-4**			73,4/6,0	73,1/6,0		73,4/6,0
**CβHβ in γ-acylated β-O-4**			78,4/4,9	78,4/4,9		78,4/4,9
**α carbon in O-ethyl substituted β-O-4**		79,7/4,5		79,1/4,6		
**C_2/H_2 α-L-arabinofuranose**					81,7/3,8	
**CβHβ in β-O-4**	84,0/4,3	83,4/4,3			83,7/4,3	
**CαHα in resinol**	85,0/4,6	85,0/4,6			85,0/4,6	84,0/4,8
**CαHα Phenyl coumaran**	86,7/5,5	86,7/5,5	86,4/5,5	86,4/5,6	86,7/5,5	86,7/5,5
**C_1-H_1 α-L-arabinofuranose**					108,0/4,8	
**3-O-acetyl- β -D xylopyranoside**	101,6/5,4					
**Aceto vanillane C_2/H_2**	111,6/7,5	111,6/7,5				
**Vanilline C_2/H_2**	110,3/7,4	111,0/7,5				
**C_2/H_2 in G units**	110,3-111,6/6,9-7,5	110,3-111,6/6,6-7,4	110,0/7,4		111,0/6,9-7,3	
**C_2 or C_5 in acetate G units**			110,0-111,63/7,0-7,6	111,3-111,6/7,0-7,6		111,3-111,6/7,0-7,6
**C_5/H_5 in G units**	115,0/6,8	115,0-115,3/6,7			115,0/	
**C_6/H_6 in G units**	119,3/6,8-7,0	119,6/6,8			118,9/6,8	
**C_3/C_6 H_3/H_6 from G unit**			121,6-121,93/6,9-7,1	119,9-121,9/6,7-7,0		121,3-121,9/7,0-7,2
**Cinnamyl alcohol**	120,3/5,3	120,3/5,3				
**Quinones**	122,3/5,7	122,3/5,7				
**CβHβ in cinnamyl aldehyde**	126,3/6,8	126,3/6,8				
**C_2/H_2 and C_6/H_6 in H units**	127,6/7,3	127,9/7,3	127,6/7,3	127,6/7,3	127,9/7,1	127,6/7,3
**Stilbenes**	127,9/7,2	128,2/7,2				

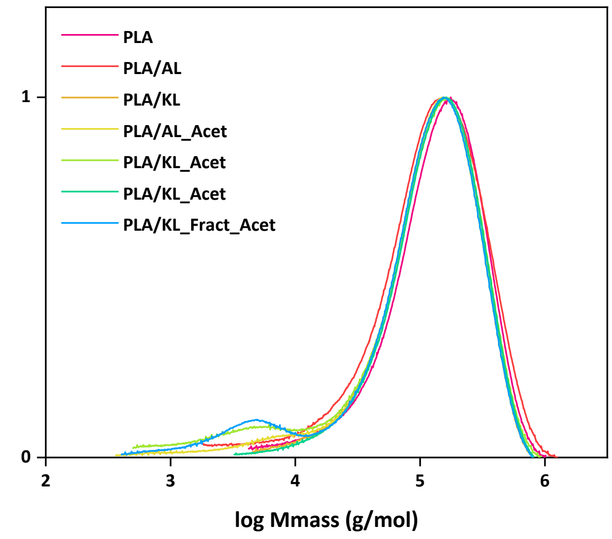

Figure S2. Molecular weight distributions of original PLA and PLA/lignin films obtained with SEC Chloroform.

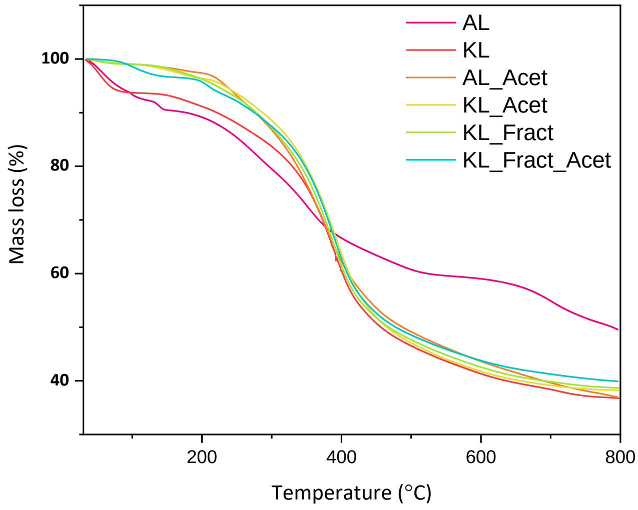

Figure S3.Thermogravimetric analysis of PLA and the different PLA/lignin films

Table S2. Average molecular weights, M_n, M_w and M_p of PLA and chloroform soluble fractions of original and 30 days hydrolytically aged PLA and PLA/lignin films.

	**Original (g/mol)**			**Day 30 (g/mol)**		
	M_n	M_w	M_p	M_n	M_w	M_p
**PLA**	90,700 ± 1,700	177,500 ± 3,100	172,000 ± 3,400	1,520 ± 50	4,470 ± 130	3,380 ± 150
**PLA/AL**	64,600 ± 3,100	171,000 ± 3500	150,000 ± 6,100	3,080 ± 150	6,400 ± 100	4,920 ± 8
**PLA/KL**	85,300 ± 3,400	160,600 ± 5,600	153,700 ± 6,300	2,180 ± 50	8,290 ± 760	3,500 ± 40
**PLA/KL-Fract**	84,300 ± 900	165,700 ± 1,500	161,800 ± 2,600	3,970 ± 40	7,630 ± 130	4,790 ± 60
**PLA/AL-Acet**	41,000 ± 1,500	155,000 ± 1,900	154,300 ± 5,500	3,680 ± 360	6,570 ± 20	4,970 ± 270
**PLA/KL-Acet**	31,400 ± 1,700	161,300 ± 2,900	166,000 ± 9,800	3,830 ± 290	7,150 ± 130	4,710 ± 70
**PLA/KL-Fract-Acet**	19,500 ± 630	142,600 ± 1,935	144,300 ± 6,600	4,500 ± 0	15,560 ± 0	4,610 ± 170

Table S3. Thermal stability as determined by $T_{5\%}$ and T_{max} of original lignin materials

Sample	$T_{5\%}$	T_{max}
**PLA (film)**	332 ± 2	364 ± 4
**AL**	138 ± 3	353 ± 1
**AL-Acet**	238 ± 1	378 ± 0
**KL**	246 ± 1	388 ± 2
**KL-Acet**	232 ± 6	394 ± 2
**KL-Fract**	207 ± 14	388 ± 0
**KL-Fract-Acet**	204 ± 7	386 ± 3

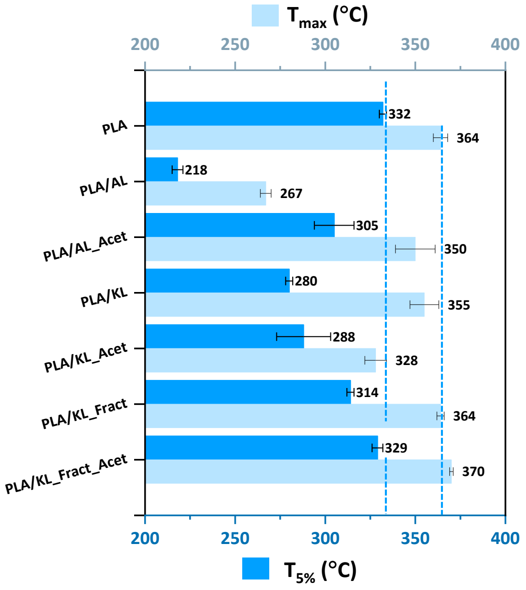

Figure S4. Thermal stability presented by $T_{5\%}$ and T_{max} of original PLA and PLA/lignin films as determined by TGA.

The thermal stability of the PLA/lignin films was influenced by the interplay between lignin composition, hydroxyl substitution and molecular weight. The effect of different lignin fractions on the thermal properties was also evaluated. All the films have been thermally degraded and the degradation onset temperature at 5% weight loss ($T_{5\%}$) and the temperature at maximum degradation rate (T_{max}) were determined from the TGA thermograms (***Figure S3***). All films had lower thermal stability compared to neat PLA, except PLA/KL-Fract-Acet, which is explained by the increased compatibility between PLA and KL-Fract-Acet and increased PLA/lignin interface. The aromatic structure of lignin is stabilized due to π-π stacking and the resonance of the unpaired electrons in hydroxyl groups, which can confer higher thermal stability compared with PLA.[2] However, the lignin materials used in this work had larger range of degradation temperatures, which explains partially the decrease of $T_{5\%}$ and

T_{max} for all films (***Figure S4, Table S3***). In addition, the increase of lignin content in PLA films, similar to this work, has been reported to decrease the thermal stability due to poorer dispersion of temperature caused by lignin aggregation in the PLA matrix.[3] PLA/AL showed the highest decrease of thermal stability, in comparison to neat PLA. Similar results were reported for lignin with high sulfonate content.[4] PLA/KL-Fract showed a smaller decrease in thermal stability compared to PLA/KL. Both PLA/AL-Acet and PLA/KL-Fract-Acet presented higher thermal stability than the original materials, due to increase in PLA/lignin interface. On the contrary, PLA/KL-Acet showed lower T_{max} compared to PLA/KL.

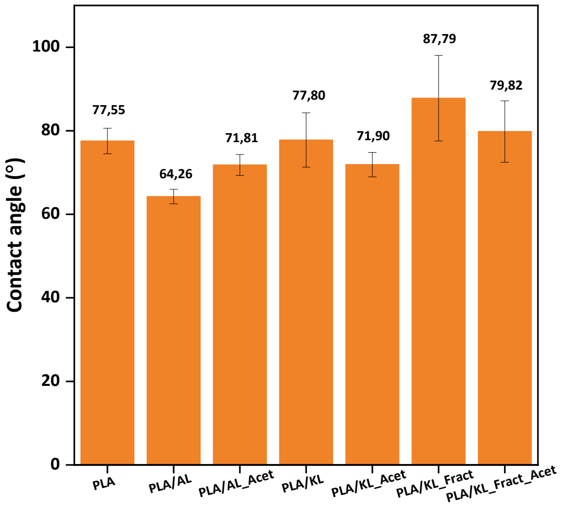

Figure S5. WCA for original PLA and PLA/lignin films

The changes in WCA depend on multiple parameters such as type of lignin, its placement in the films and surface morphology and roughness, where the effect of the latter was more pronounced. PLA is considered hydrophobic, however the introduction of AL, a water-soluble lignin, decreased the contact angle more than 10°. The addition of KL did not change the water contact angle, which is explained by the presence of the majority of the lignin close to the bottom surface, as indicated by FE-SEM. An increase in water contact angle was seen for PLA/KL-Fract and PLA/KL-Fract-Acet, likely caused by the high rugosity of the surface.[5] On the contrary, acetylated lignin in PLA/AL-Acet and PLA/KL-Acet decreased the water contact

angle, which typically indicates increased hydrophilicity. However, as lignin acetylation is expected to increase the hydrophobicity of lignin, this could be caused by the flat surface patterns shown by FE-SEM.

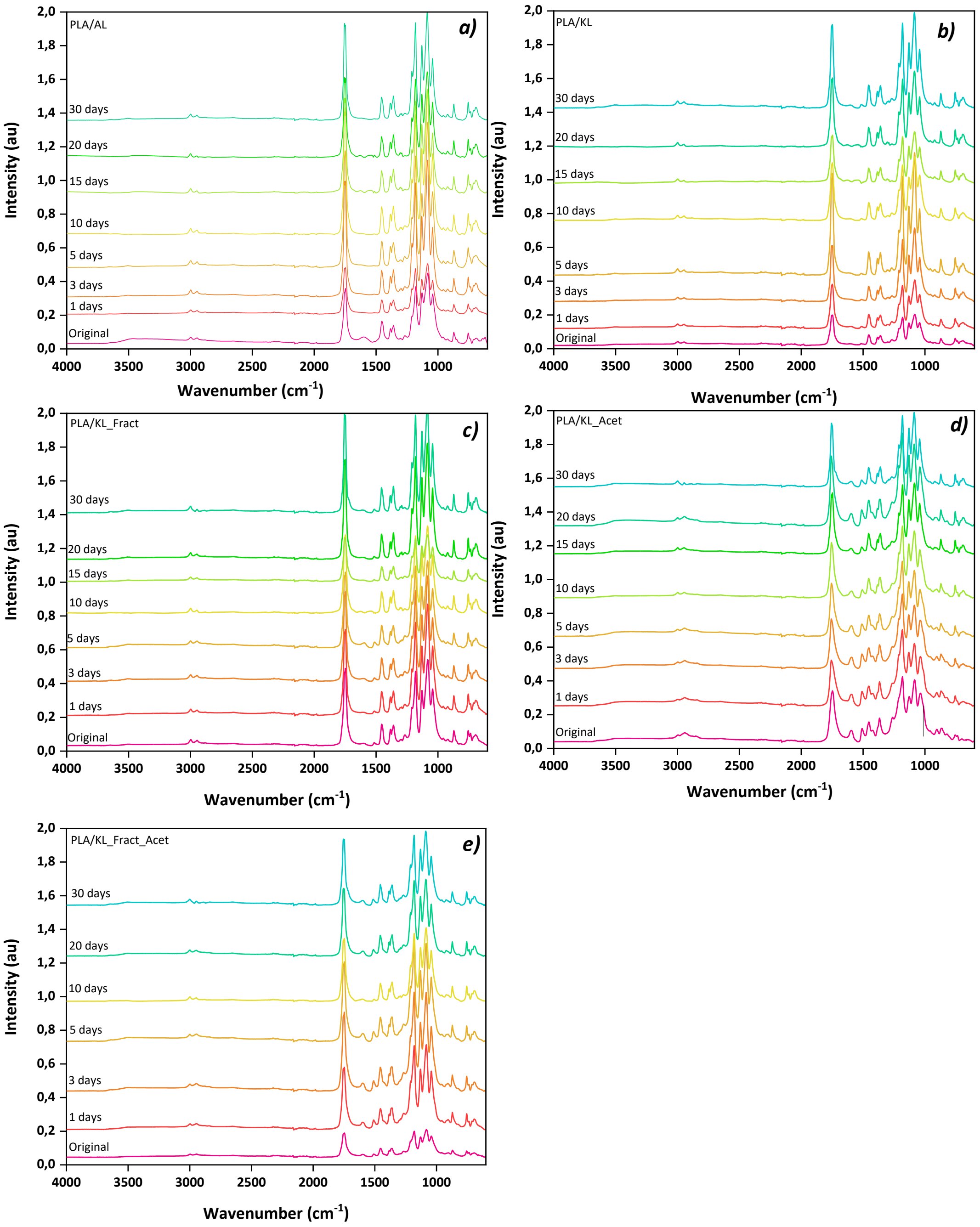

Figure S8. Evolution FTIR spectrum during hydrolytic degradation for a) PLA/AL, b) PLA/KL, c) PLA/KL-Acet, d) PLA/KL-Fract and e) PLA/KL-Fract-Acet.

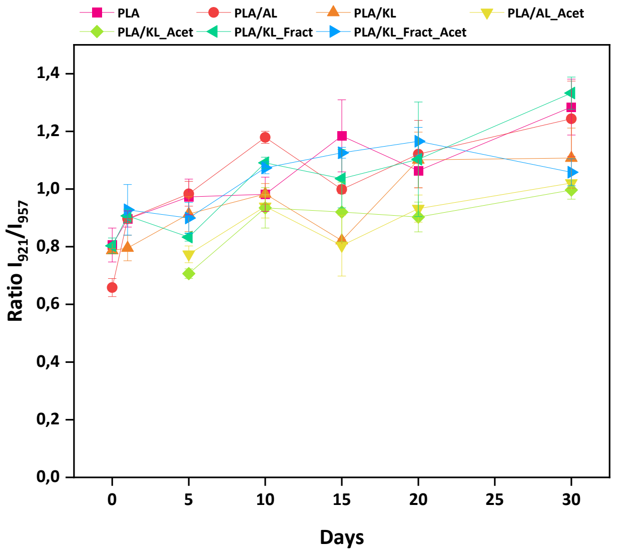

Figure S9. Evolution of the intensity ratio of peak 921 cm^{-1} and 957 cm^{-1} during hydrolytic degradation of PLA and PLA/lignin degraded samples.

Table S4. Assignments of C/H signals of lignin units in all original and aged at 30 days PLA/lignin films, detected by 2D HSQC spectroscopy in DMSO-d_6.

Signal description	δ_C (ppm)	δ_H (ppm)
CH_3 PLA	15,7-16,6	1,4-1,8
CH_3 end PLA	19,7-20,2	1,2-1,3
O-acetylated	19,7-20,6	1,9-2,3
C_β/H_β acetylated aryl propanol	29,5-30,0	1,9-2,1
C_β/H_β cinnamyl alcohol	34,2-35,7	1,7-1,7
C_α/H_α secoisolariciresinol	33,5-33,9	2,5-2,6
C_α/H_α cinnamyl alcohol	30,4-31,2	2,5-2,6
C_β/H_β secoisolariciresinol	42,2-42,5	1,9-1,9
C_β/H_β phenylcoumaran β5	53,1	3,5
Methoxy group (O-CH_3)	55,0-55,5	3,8-3,8
C_β/H_β resinol ββ	53,8	3,1-3,1
C_γ/H_γ aryl ether βO4	59,6-60,1	3,4-3,6
Ethyl ethers from substitution of the α carbon in βO4	63,3-63,8	3,3-3,4
Ethyl ethers from O-alkylation of phenolic OH	61,1	3,8
CH_2 in ester from condensation of acid and alcohol from lignin or ethanol	59,3-59,8	4,0-4,1
C_γ/H_γ phenylcoumaran β5	62,8-63,4	3,3-3,7
C_γ/H_γ aryl ether βO4 acetylated	62,6-64,1	4,0-4,3
C_γ/H_γ cinnamyl alcohol	63,1-63,4	3,9-3,9
C_γ/H_γ cinnamyl alcohol acetylated	64,1	4,7
CH- end PLA	65,4-65,8	4,1-4,2
CH PLA	68,1-69,1	4,9-5,2
C_γ/H_γ resinol ββ	70,7-71,1	3,7-4,2
C_α/H_α aryl hydroxy acetic acid	73,7-74,4	4,4-4,8
C_α/H_α aryl ether βO4	71,1-71,4	4,7-4,8

C_α/H_α aryl ether βO4 ethyl	79,7-80,1	4,5-4,5
β-D-xylopyranoside	72,4-75,4	3,1-3,5
C_α/H_α aryl ether βO4 acetylated	74,1	6,0-6,0
C_β/H_β aryl ether βO4	83,7-84,0	4,3-4,3
C_β/H_β aryl ether βO4 acetylated	78,7	4,8-4,9
C_α/H_α resinol ββ	84,4-85,0	4,6-4,8
C_α/H_α phenylcoumaran β5	86,7-87,0	4,6-5,5
C_2/H_2 guaiacyl unit	109,6-112,3	6,8-7,1
C_5/H_5 guaiacyl unit	115,0-115,3	6,7-7,1
C_3/H_3 and C_6/H_6 guaiacyl unit acetylated	119,1-122,6	6,8-7,1
C_6H_6 guaiacyl unit	119,3-120,3	6,7-7,0
Stilbene B1	127,4-128,2	7,1-7,3
Stilbene B5	122,9	7,5-7,5
C_β/H_β cinnamyl aldehyde	126,3-126,9	6,8-7,6

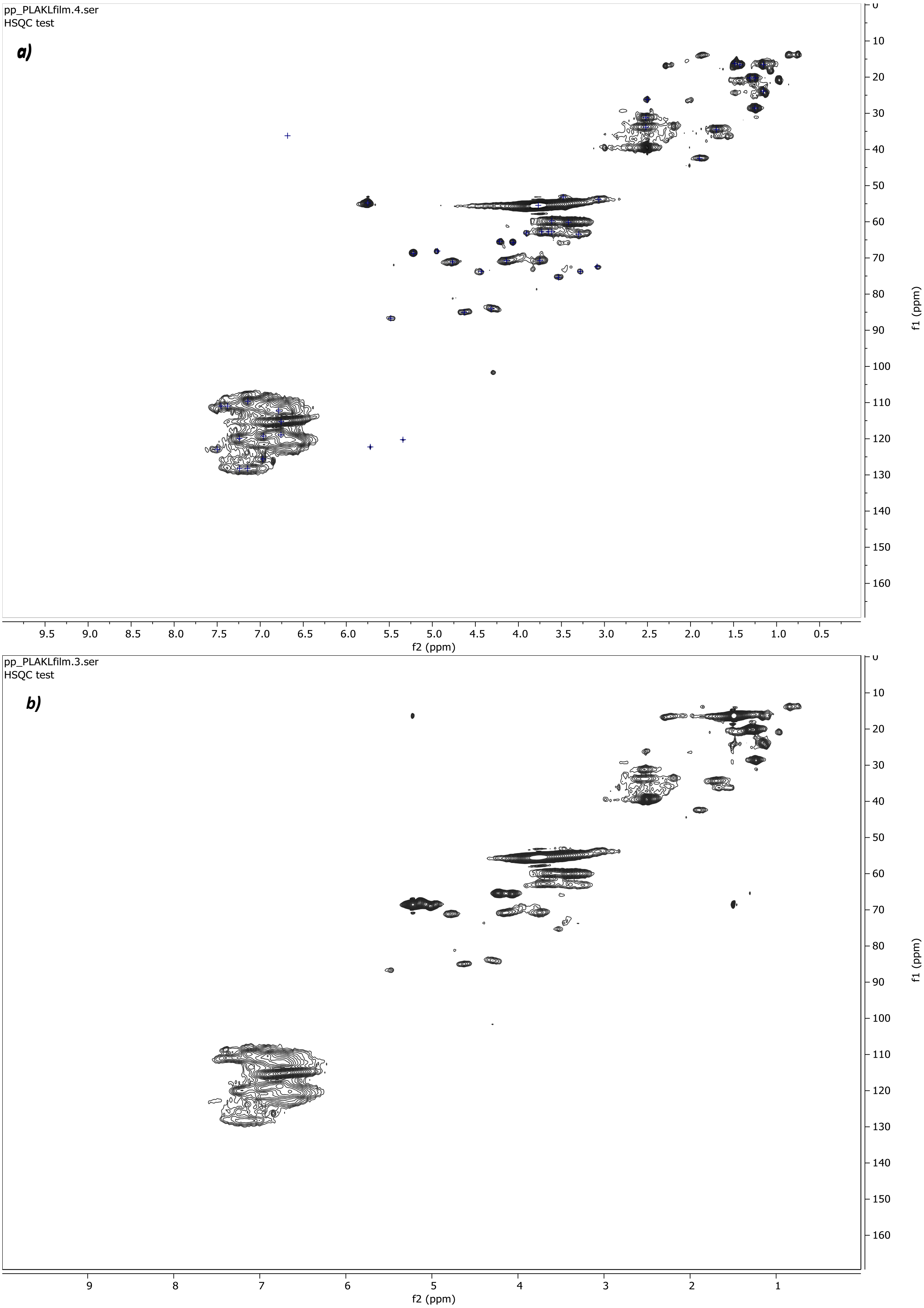

Figure S10. 2D-NMR HSQC spectrum of a) original PLA/KL and b) aged PLA/KL at day 30 under hydrolytic degradation.

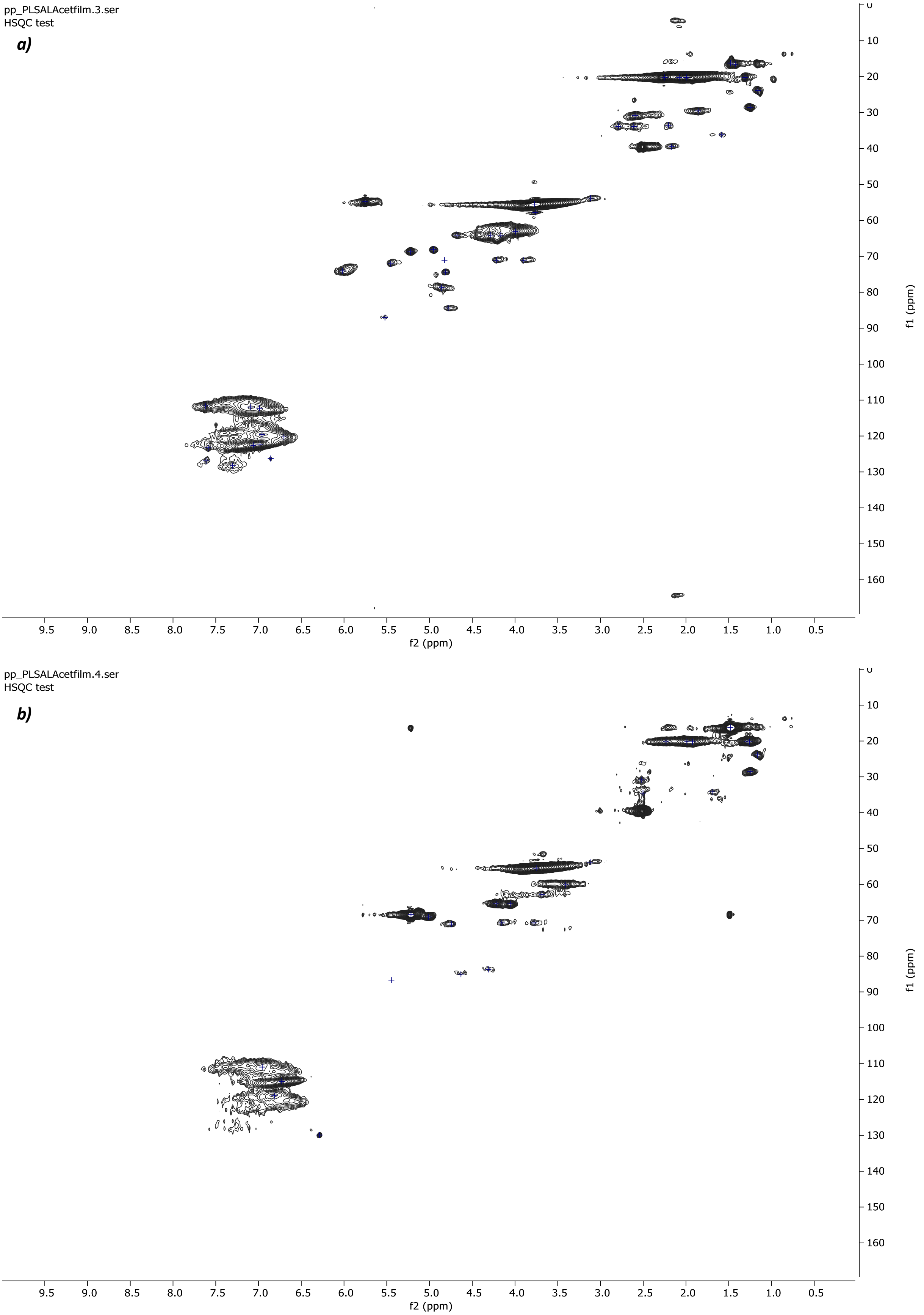

Figure S12. 2D-NMR HSQC spectrum of a) original PLA/AL_Acet and b) aged PLA/AL_Acet at day 30 under hydrolytic degradation.

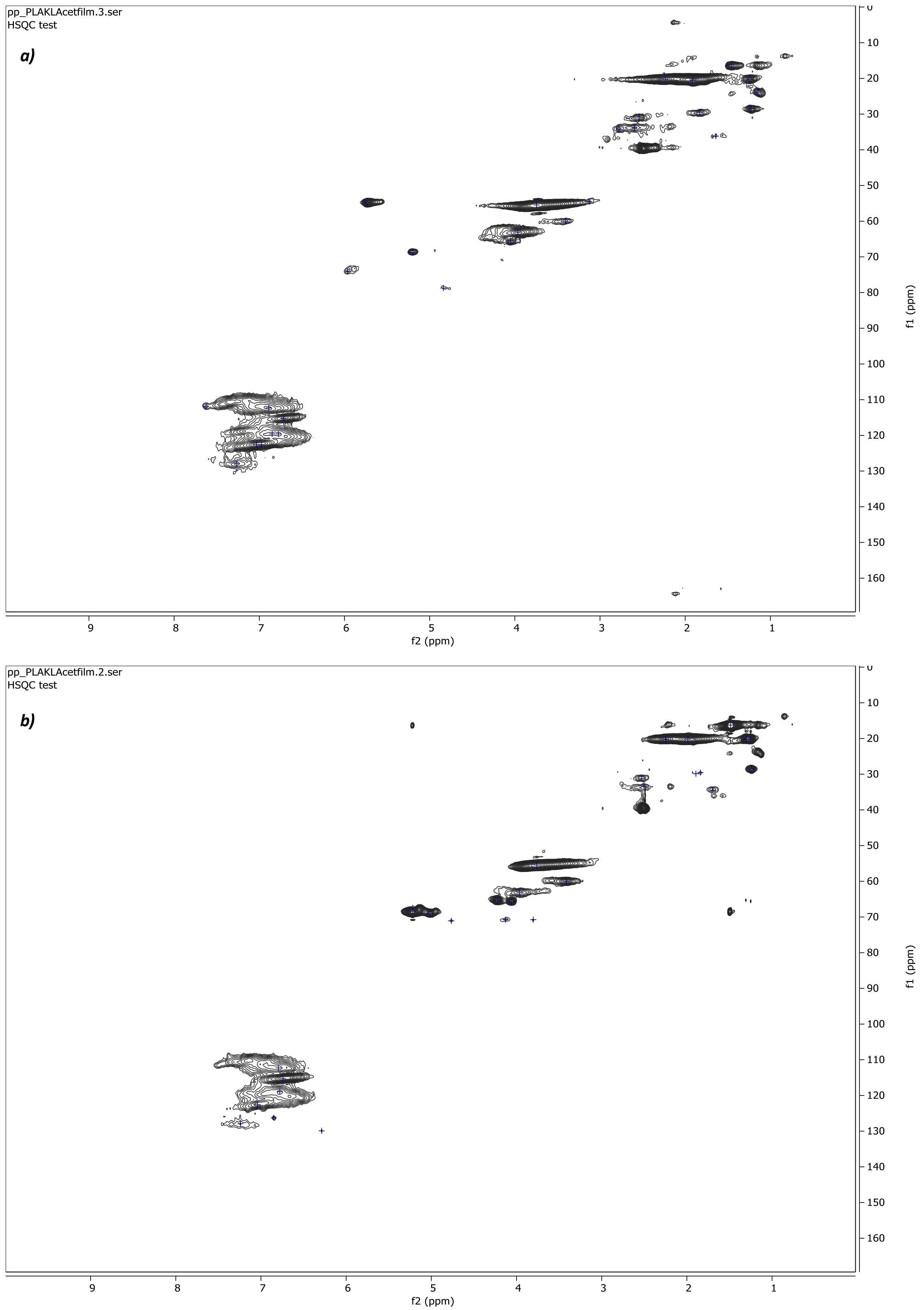

Figure S14. 2D-NMR HSQC spectrum of a) original PLA/KL_Acet and b) aged PLA/KL_Acet at day 30 under hydrolytic degradation.

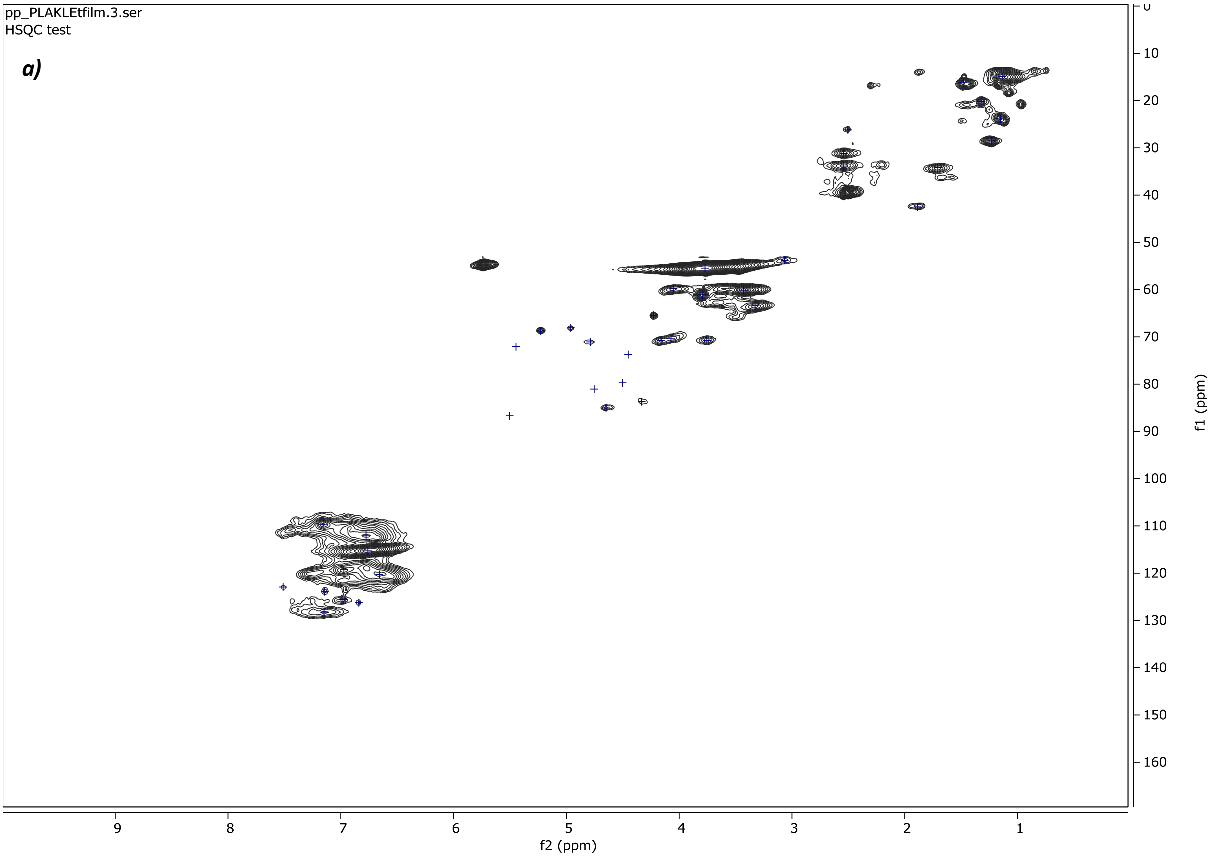

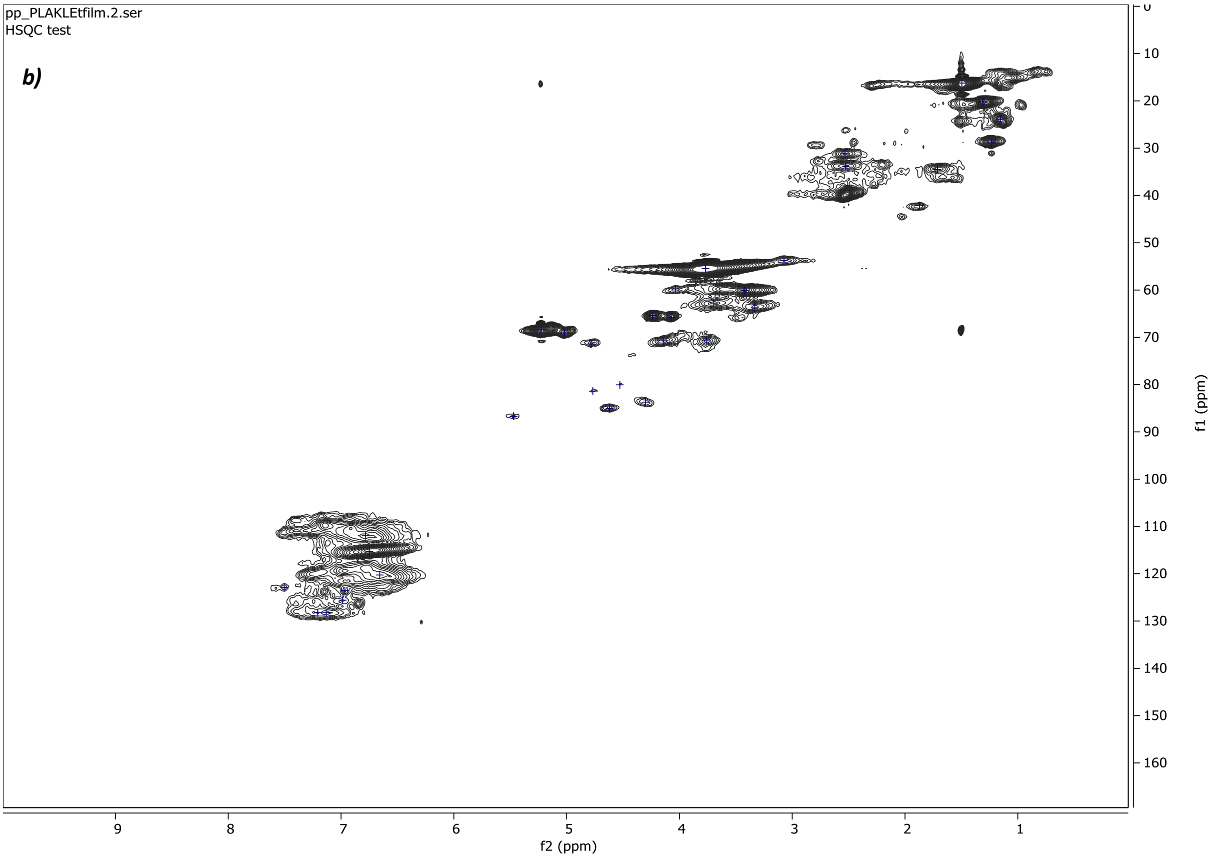

Figure S16. 2D-NMR HSQC spectrum of a) original PLA/KL_Fract and b) aged PLA/KL_Fract at day 30 under hydrolytic degradation.

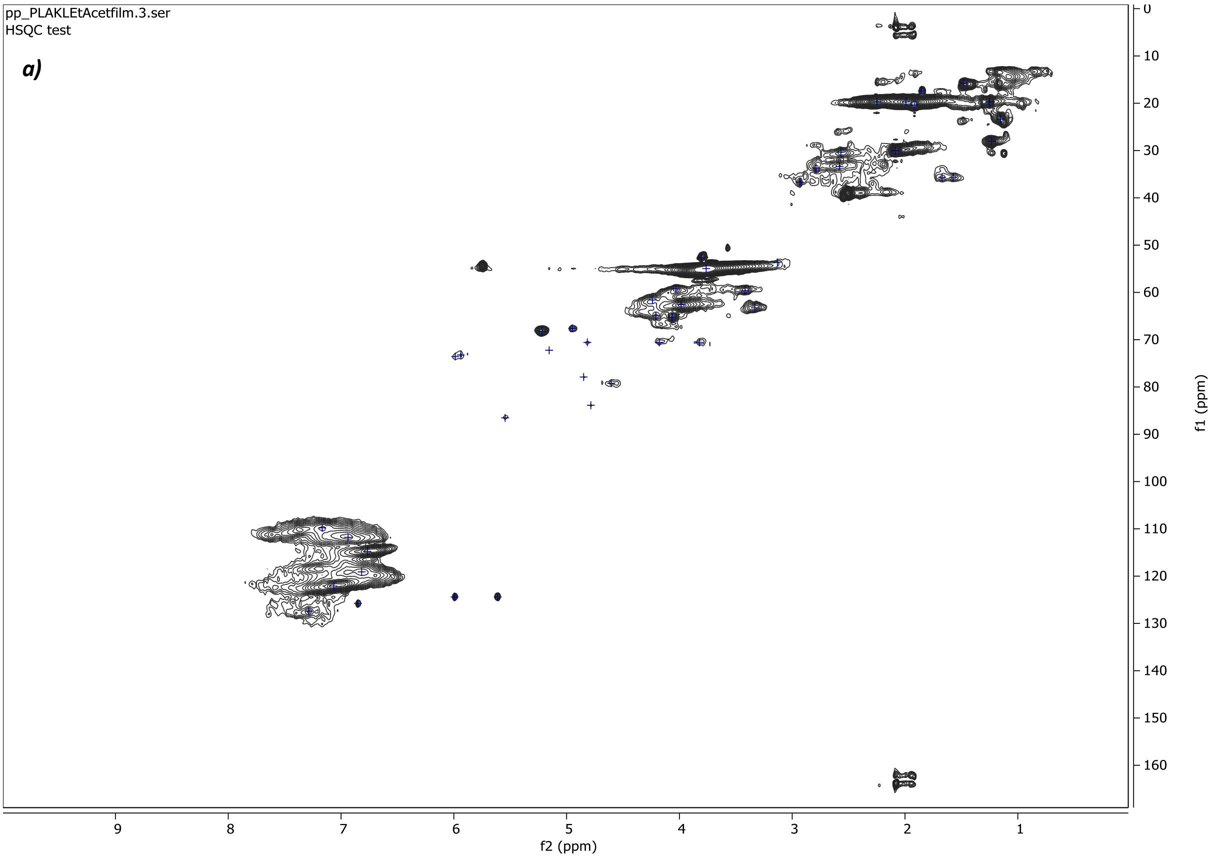

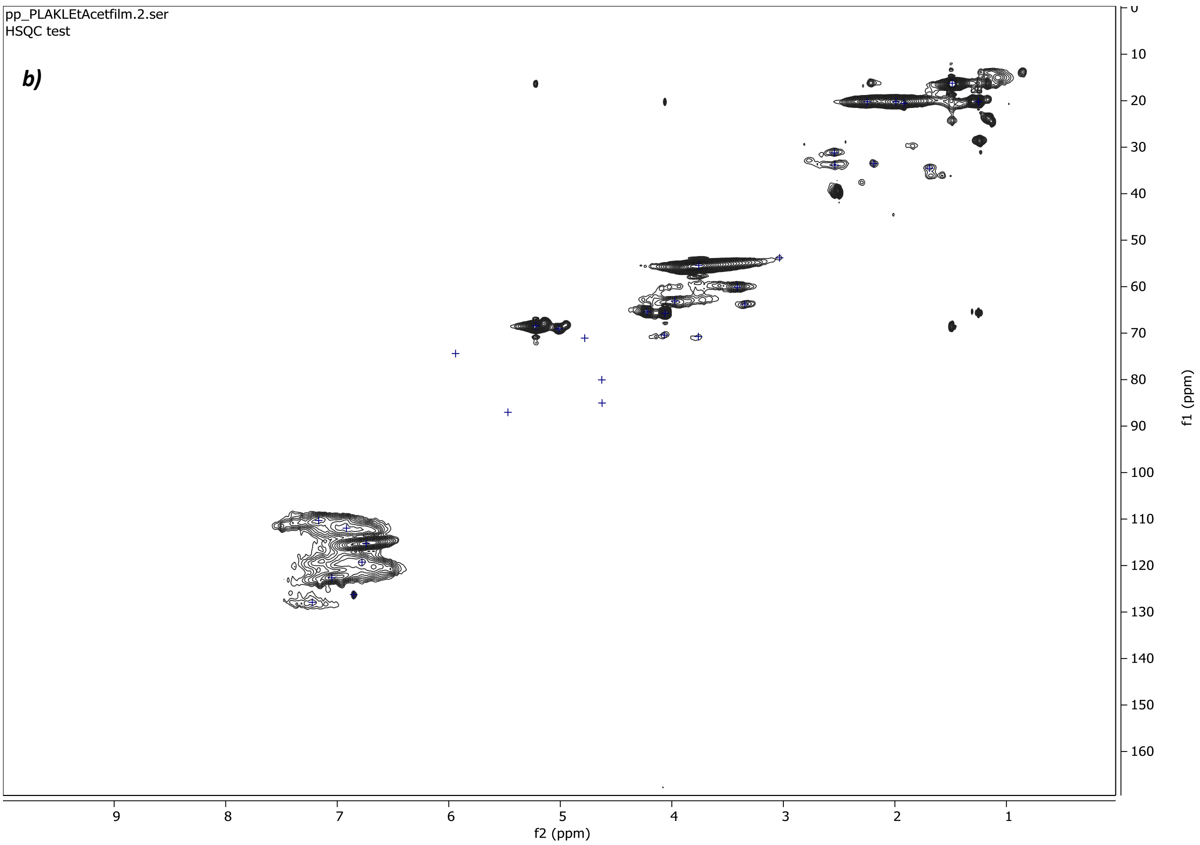

Figure S18. 2D-NMR HSQC spectrum of a) original PLA/KL_Fract_Acet and b) aged PLA/KL_Fract_Acet at day 30 under hydrolytic degradation.

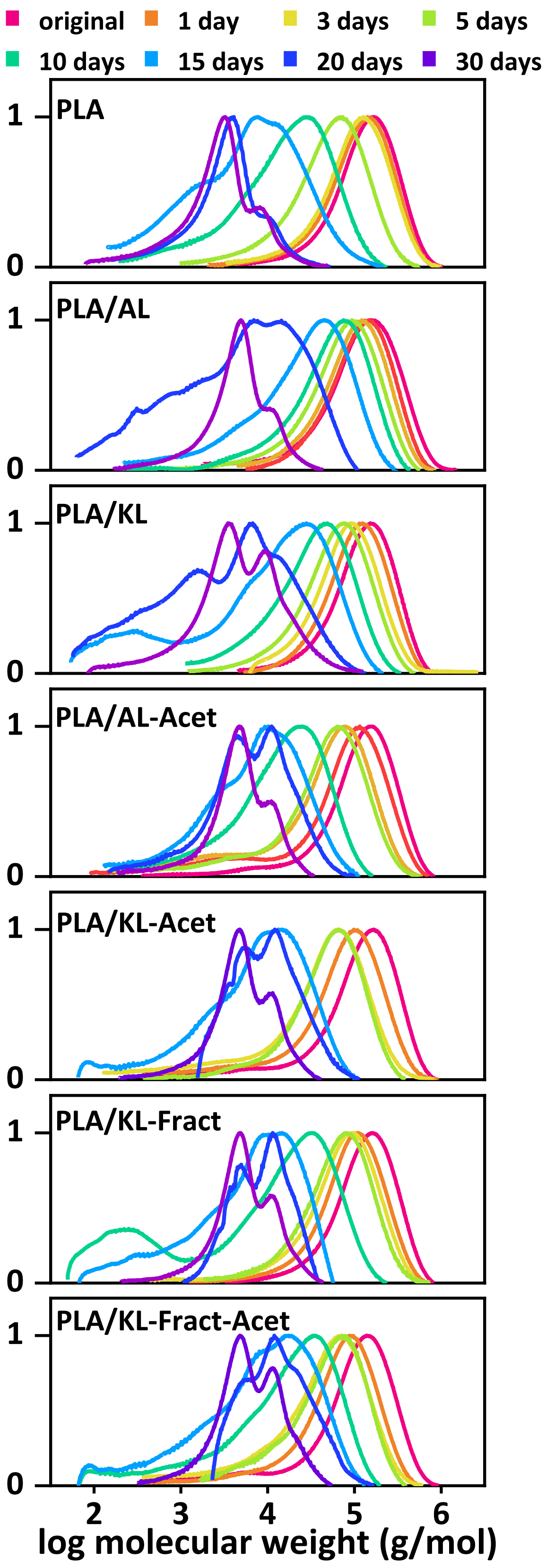

Figure S20. Evolution of molecular weight distributions of PLA and PLA/lignin samples during hydrolytic degradation obtained with SEC analysis using the chloroform soluble fraction.

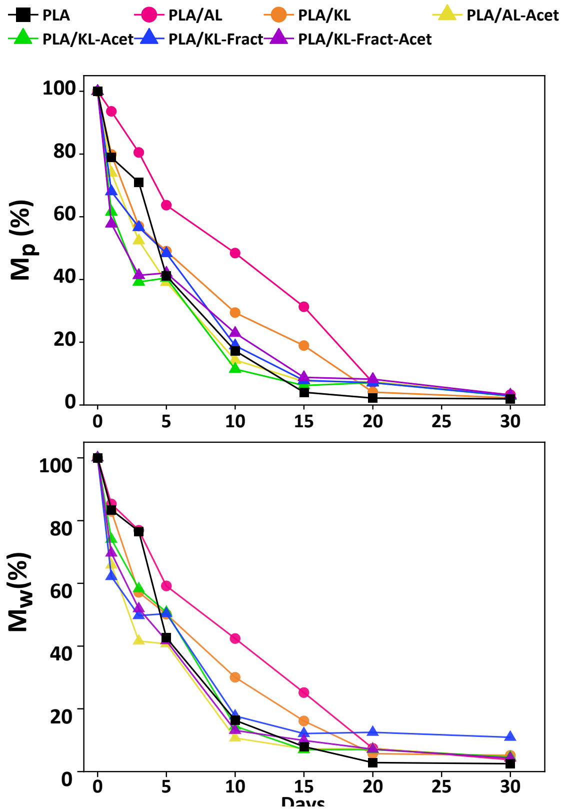

Figure S21: Evolution of molecular weight of the chloroform soluble fraction of PLA and PLA/lignin samples during hydrolytic degradation: a) M_p and b) M_w.

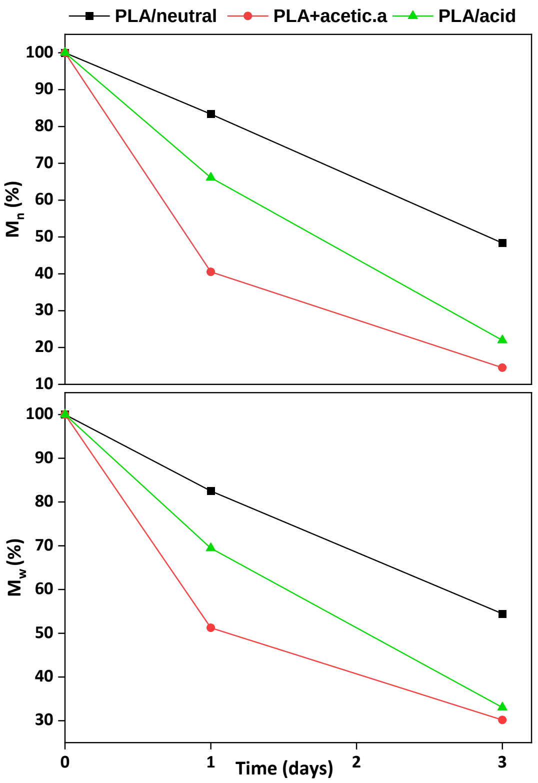

Figure S22: Evolution of molecular weight of PLA/neutral, PLA/acid and PLA/+acetic.a samples during hydrolytic degradation with SEC Chloroform: a) M_n and b) M_w.

Three control experiments were performed to confirm the accelerating influence of released acetic acid on the hydrolytic degradation rate of PLA. The decrease in molecular weight (M_n and M_w) for the three conditions tested (***Figure S13***) was seen to clearly depend on the experimental conditions and the addition of acetic acid to the surrounding medium or embedment in the films. Both PLA/acid and PLA+acetic.a showed a higher decrease of molecular weight than neat PLA, due to the catalytic effect of the acid on the cleavage of ester bonds. However, PLA+acetic.a illustrated the highest decrease of molecular weight due to the presence of the acetic acid embedded in the PLA film rather than in the surrounding medium. These findings support the hydrolysis accelerating effect of acetic acid released from PLA/lignin materials with acetylated lignin.

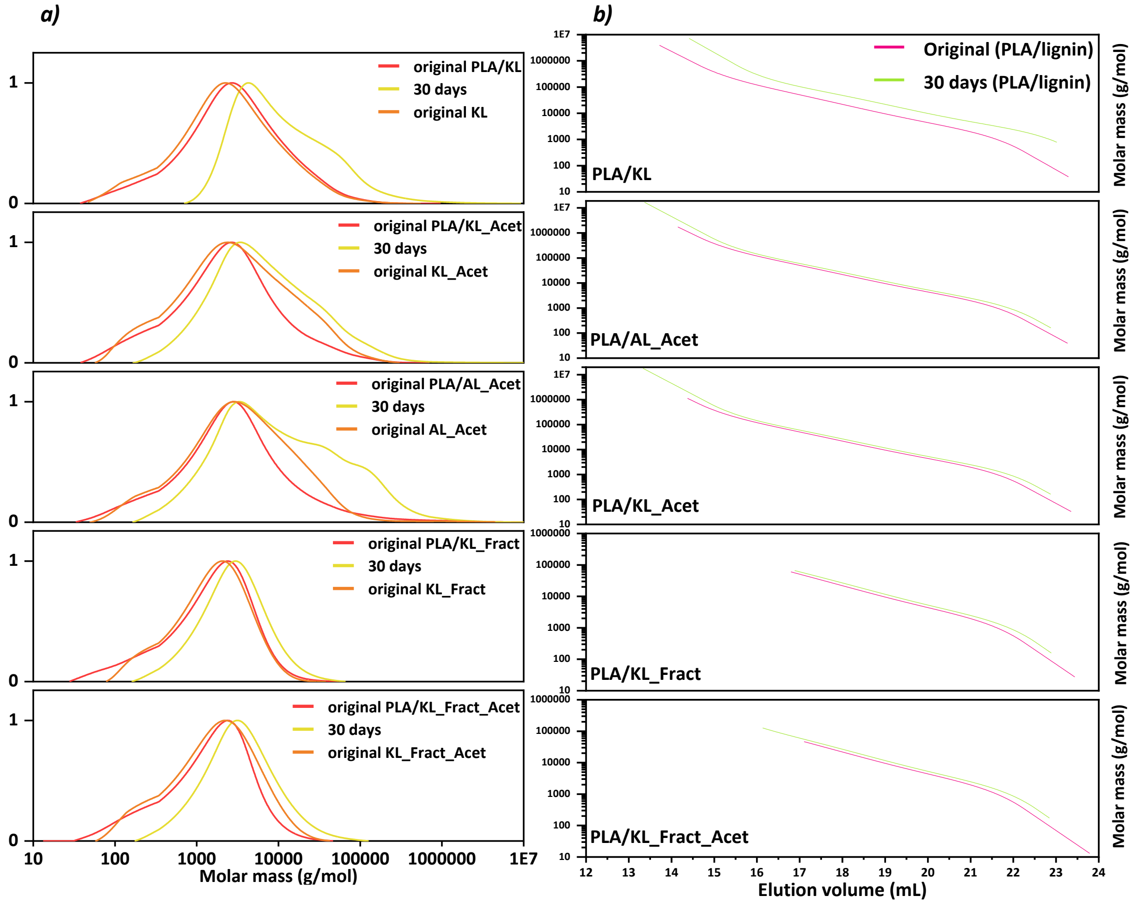

Figure S24. a) Evolution of molecular weight distributions of DMSO/ LiBr soluble fractions in PLA/lignin films during hydrolytic degradation with SEC and b) Molecular weight vs elution time for all original PLA/lignin films (except PLA/AL) and hydrolytically aged films at day

Pristine lignin and lignin from PLA/lignin films have been compared to understand if the lignin fraction would be affected by the solvent casting or by the PLA. It is indicated in the results how a fraction of

all acetylated lignin, stay embedded in the PLA matrix during dissolution in DMSO/LiBr (***Figure S14a***). This fraction appears to be of high molecular weight and is probably due to strong interactions between highly acetylated lignin and PLA matrix. In addition, after 30 days of degradation, all DMSO/LiBr soluble fractions present an increase in molecular weight, especially for PLA/KL, PLA/AL-Acet and PLA/KL-Acet. Since the molecular weight of PLA/KL-Fract and PLA/KL-Fract-Acet is fairly constant throughout the degradation experiment this could indicate a relationship between the increase of the molecular weight and the molecular weight of the pristine lignin. The analysis of PLA/AL-Acet and PLA/KL-Acet with HSQC spectroscopy revealed partial deacetylation and a decrease in βO4 linkages, which would explain partially the increase of molecular weight between original PLA/lignin films and films aged at day 30, due to the loss of compatibility with PLA, and the easier dissolution of the lignin in DMSO/LiBr. However, the extent of the increase in KL-Acet and AL-Acet is not explained, similar to the increase seen for KL. To understand better the changes between the original lignin and the aged sample, comparison of the molecular weight vs elution volume has been performed, ***Figure S14b***. Analyzing the curves from PLA/KL, it is seen how both lignin have similar elution times, but for day 30 the molecular weight is greater than the original sample. Therefore, the size of the lignin units that passes through the columns is similar, but the molecular weight is higher. This could mean that the KL at day 30 is denser than original KL, which could indicate aggregation of lignin. Lignin aggregation is a result of weak inter- and intra-molecular forces derived from the many functional groups and units presents in lignin, such as hydroxyl or aromatic units. Water is a very weak solvent for Kraft lignin, and therefore lignin molecules tend to be more compact in this medium. However, other factors such as branching or the molecular weight are thought to have a role in chain entanglement, and therefore aggregation characteristics.[6] It is therefore plausible that after 30 days in water and PLA medium, KL has aggregated forming denser units, resulting in measured higher molecular weight from SEC. This effect is not as pronounced for PLA/KL-Fract, since both curves are rather similar. The decrease in molecular weight of the lignin seems to hinder the aggregation. In addition, for this sample is easier to see how a fraction of the smaller molecular weight lignin is not present after 30 days, indicating possible release in the water medium. For PLA/KL-Acet and PLA/AL-Acet, the curve shapes are similar. A small molecular weight fraction from the original sample is not

present at day 30 and at lower elution volumes, a high molecular weight fraction appears with "bigger" size than present in the original sample. Lignin molecular structure and the complexity of inter and intra molecular interactions pose difficulties when determining the molecular weight via SEC. However, it is known that different lignin molecules within lignin can be separated by means of different solvents, due to differences in their molecular structure and weight, a process called fractionation.[7] We believe that the deacetylation reactions of the many different lignin molecules within AL-Acet and KL-Acet during degradation caused changes in the inter and intra molecular interactions of such molecules, causing them to behave differently when solubilized in DMSO/LiBr as opposed to the original lignin. The combination of the different deacetylated lignin molecules and loss of certain βO4 rich lignin fractions could have yield the seen molecular weight distributions. On the other hand, PLA/KL-Fract-Acet shows again differences at low molecular weight fraction, similar fractions in the middle zone of the elution times, and a high molecular weight fraction at higher elution times not present in the original sample.

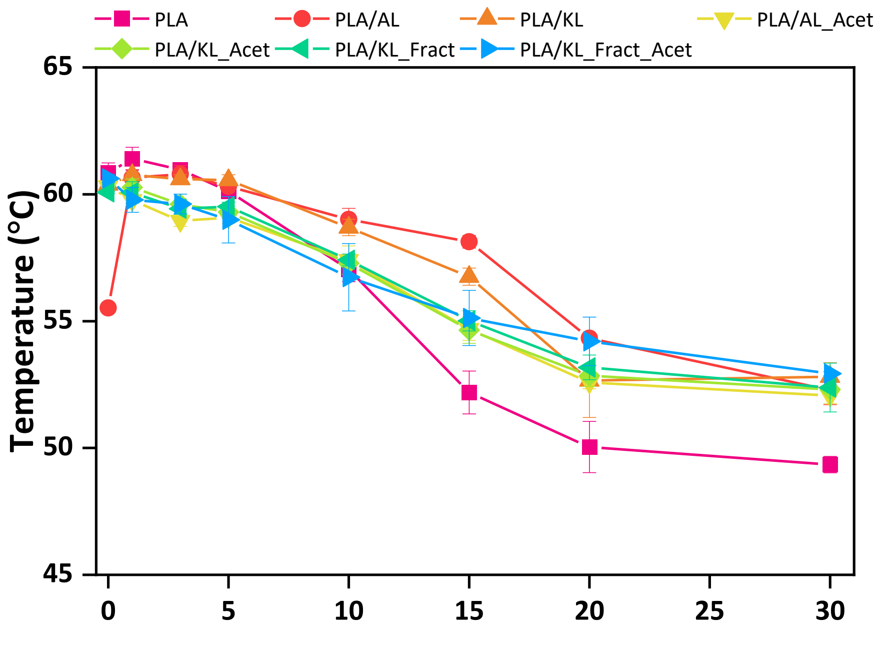

Figure S25. Evolution of T_g during hydrolytic degradation of all PLA and PLA/lignin films.

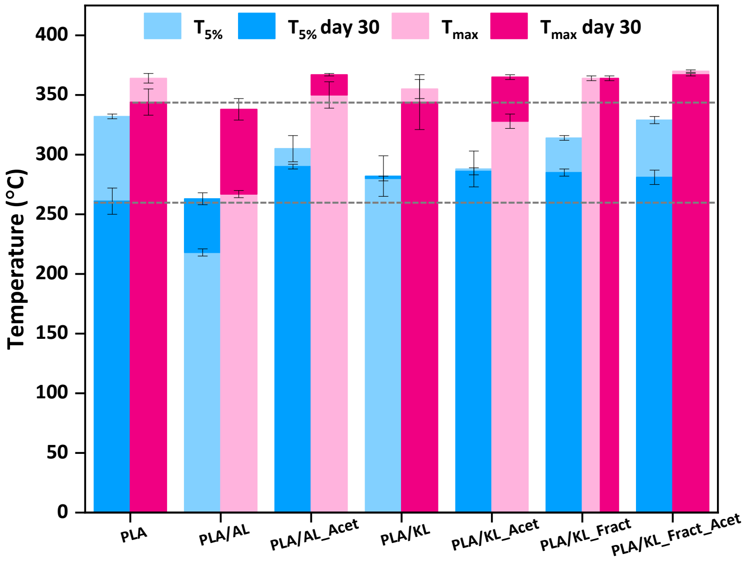

Figure S26. Thermal parameters, $T_{5\%}$ and T_{max}, of original and aged PLA and PLA/lignin films under hydrolytic degradation at day 30.

The apparent increase of $T_{5\%}$ and T_{max} for PLA/AL only reflects the effect of the dissolution of AL in water. However, upon degradation, thermal stability is lost but not as significantly as for neat PLA. Both PLA/AL-Acet and PLA/KL-Acet show the lowest decrease in $T_{5\%}$, which can be explained by the more homogeneous dispersibility of lignin particles in PLA matrix. However, the T_{max} increase for both films after 30 days, until values similar to those of original neat PLA can be explained by the deacetylation of lignin, already confirmed by FTIR and HSQC, which would cause the increase of OH groups and therefore increase the thermal stability of lignin. PLA/KL´s $T_{5\%}$ stays constant at around 280 °C, while T_{max} decreases, being the loss of thermal stability less pronounced than for neat PLA film. The $T_{5\%}$ of PLA/KL-Fract decreases while T_{max} stays fairly constant. Finally, PLA/KL-Fract-Acet shows a decrease of $T_{5\%}$ from and an almost constant T_{max}.

Regarding the morphology of the original PLA/lignin films, the neat PLA film presented a flat surface with spherulites, while partial phase separation caused by incorporation of AL. For the PLA/KL film surface, interconnected spherulites were present. The incompatibility of KL with PLA was clear from the aggregation of KL in the cross-section in order to reduce the interface area between PLA and KL. KL-Fract also presented a heterogeneous surface with increased

rugosity with respect to neat PLA. PLA/AL-Acet and PLA/KL-Acet presented similar surface and cross-section morphologies. The increased compatibility was supported by the formation of regular round shaped lignin aggregates on the top surface, embedded in a PLA matrix, creating a flatter surface similar. The formation of this flat surface could be caused by the presence of relatively big aggregates of lignin, which might prohibit the spherulite growth process on the surface. The PLA/KL-Fract-Acet film surface was similar to PLA/KL, with interconnected spherulites, but with the presence of distributed small round lignin aggregates. From the cross-section, the even distribution of aggregates could be appreciated, indicating an increased compatibly with respect to AL-Acet and KL-Acet. This effect, in turn allowed for the formation of the spherulite interconnected domains, as seen for PLA/KL.

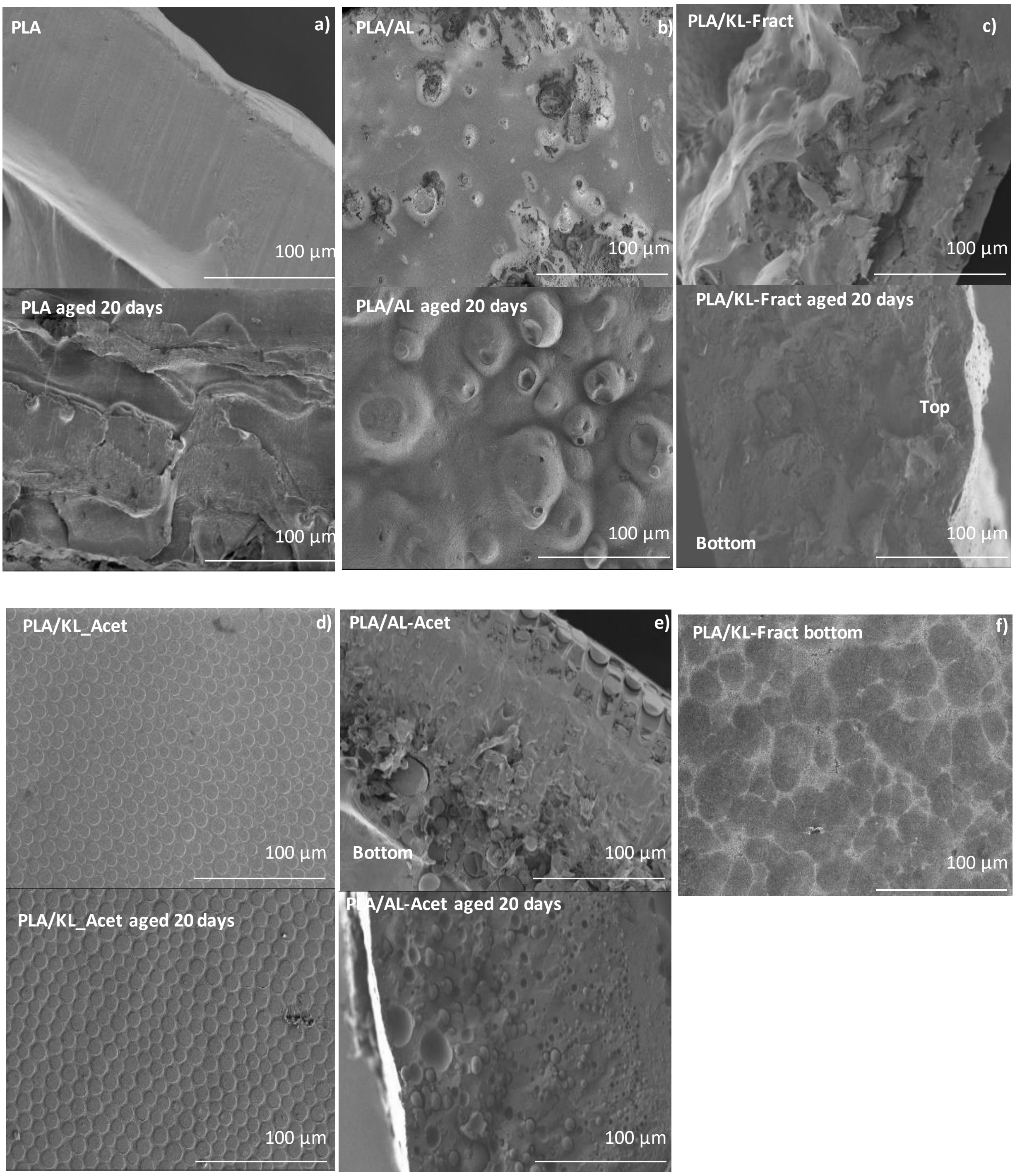

Figure S28. PLA and PLA/lignin surface and cross-section morphology for original and aged materials imaged by FE-SEM: a) PLA, b) PLA/AL, c) PLA/KL-Fract, d) PLA/KL-Acet, e) PLA/AL-Acet and f) PLA/KL-Fract bottom surface

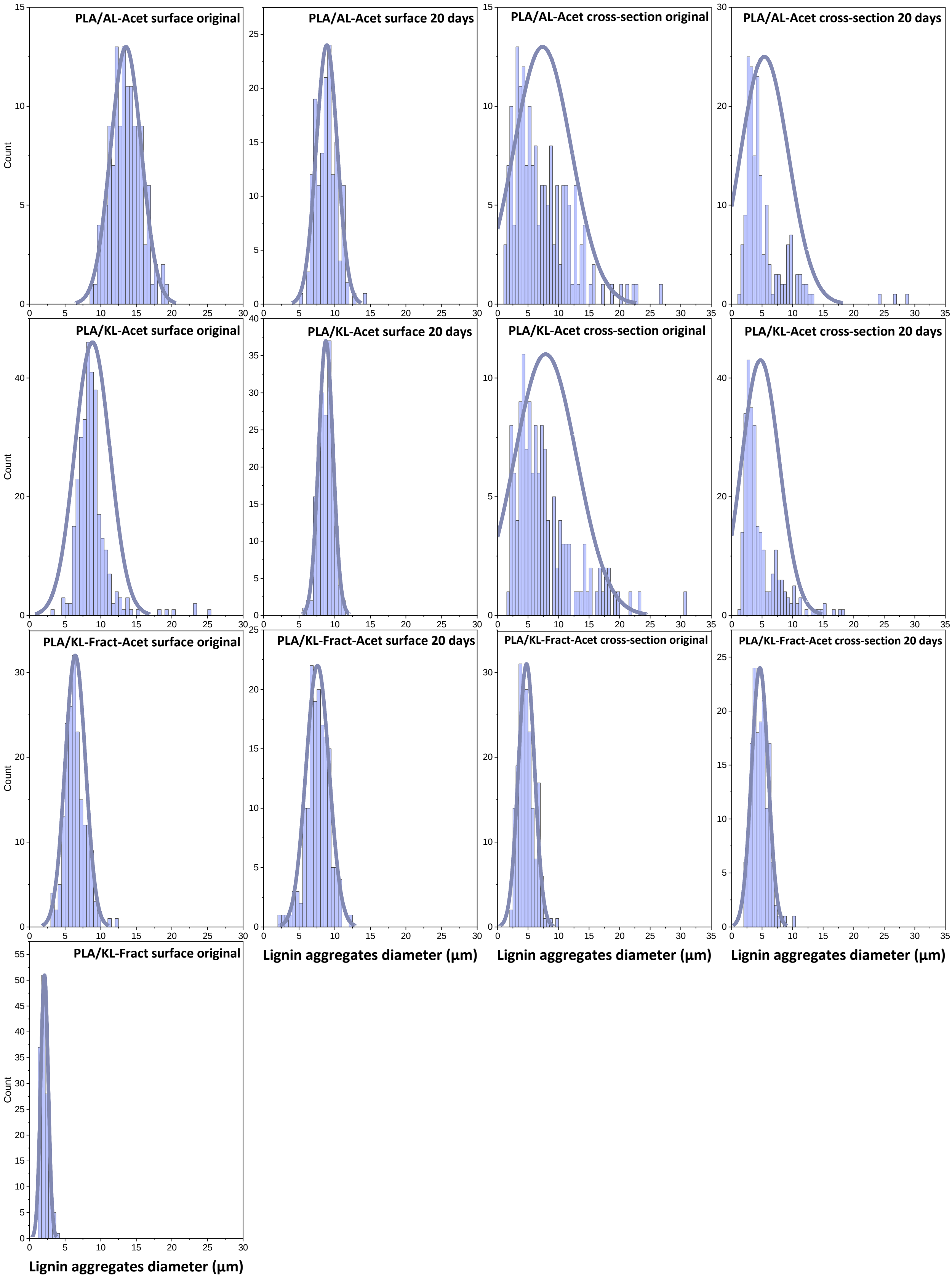

Figure S29: Distribution of lignin aggregates diameter in original and aged after 20 days PLA/lignin films.

Table S5*: Average diameter of lignin aggregates in original and aged after 20 days PLA/lignin samples.*

	Average diameter lignin aggregates (µm)			
	Surface original	Surface 20 days	Cross-section original	Cross-section 20 days
**PLA/KL**	[a]	[a]	[a]	[a]
**PLA/AL**	[a]	[a]	[a]	[a]
**PLA/KL-Fract**	2,1±0,5	[b]	[a]	[a]
**PLA/KL-Acet**	8,8±2,5	8,7±1,0	7,9±5,1	4,7±1,0
**PLA/KL-Fract-Acet**	6,4±1,4	7,6±1,6	4,7±1,3	4,6±1,4
**PLA/AL-Acet**	13,5±2,2	8,9±1,5	7,4±4,7	5,4±3,9

[a] The sizes of the aggregates is too big or the shape too heterogeneous to be analyzed

[b] After 20 days, only the remaining voids after the lignin liberation into the medium was seen

Table S6*: Particle size distribution parameters of different lignin materials in DCM at a concentration of 0.3 mg/mL.*

	D(10) (µm)	D (50) (µm)	D(90) (µm)	D(90)/D(10)
**KL**	14,10±3,30	25,95±2,25	42,65±0,75	3,19±0,69
**AL**	3,17±0,05	19,15±0,05	34,25±0,45	10,83±0,30
**KL-Fract**	53,37±1,25	72,57±2,13	97,17±3,47	1,82±0,02
**KL-Acet**	3,35±0,03	5,88±0,11	9,46±0,22	2,83±0,04
**KL-Fract-Acet**	8,71±0,72	33,97±2,43	62,10±3,25	7,15±0,20
**AL-Acet**	0,02±0,00	0,03±0,00	0,06±0,00	3,08±0,22

Lignin particle size distributions varied widely in the solvent (DCM) due to differences in their molecular weight and structure. AL and KL both show a similar particle size median D(50), however, KL has a much narrower size distribution (D(90)/D(10)) than AL. On the other hand, KL-Fract particles in DCM are the largest amongst all the lignin types. This can be caused by the higher number of OH aromatic groups, causing the lignin to have more polar centres causing more extensive aggregation in the solvent. At the same time, KL-Fract shows the narrowest distribution due to the fractionation effect, i.e., the narrower molecular weight distribution. The acetylation of the lignins causes a decrease of the particle size in all cases due

to the decreased amount of free OH groups. While KL-Acet shows a narrow size distribution, KL-Fract-Acet presents somewhat larger particles. AL-Acet shows the smallest particle sizes in DCM and a much narrower distribution. Comparing the particle sizes of the lignins in the solvent and in the final films (***Table 5***) both PLA and the lignin concentration (during film casting is 3 mg/mL rather than 0.3 mg/mL) likely influence it. For KL-Fract, we see how despite the large particle size in DCM, interactions between the lignin and PLA during solvent casting result in the formation of small aggregates on the PLA surface. On the other hand, for KL-Acet the particle size in DCM is similar to that seen on the PLA matrix. This effect is not seen for KL-Fract-Acet, where the particle size in DCM is much larger than what is seen in the PLA matrix, indicating good compatibility with PLA. On the contrary, AL-Acet aggregate size in only DCM is much smaller than in the final PLA/AL-Acet film, indicating aggregation during the solvent casting process.

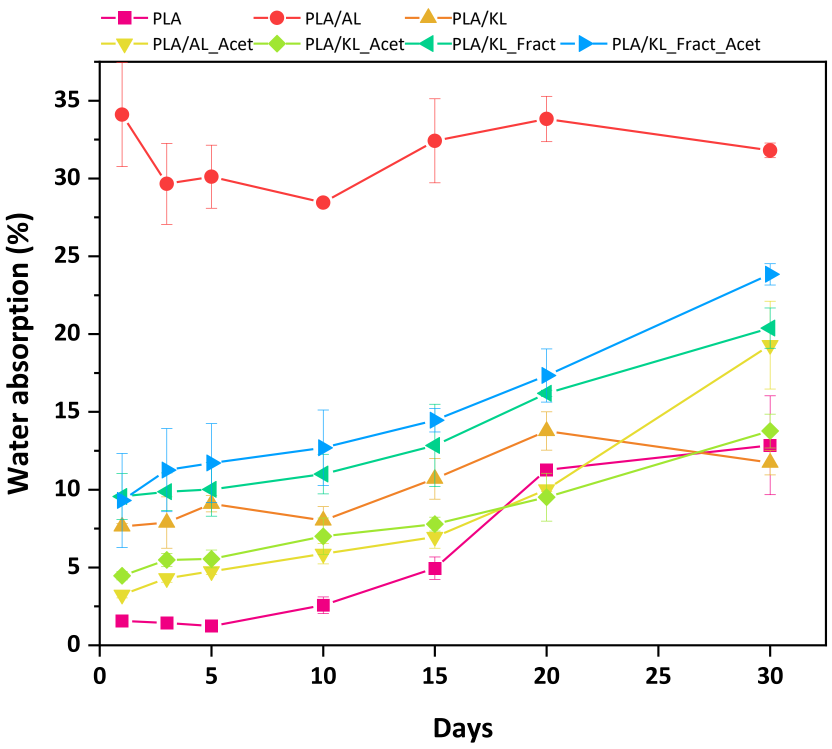

Figure S30. Evolution of water absorption during hydrolytic degradation PLA and PLA/lignin films.

Saturation of water absorption was observed for PLA, PLA/AL and PLA/KL, while a more linear increase of water absorption was found for the fractionated and/or acetylated PLA/lignin films. PLA/AL showed the highest water absorption of all films, caused by dissolution of the majority of water-soluble AL, substantially increasing the surface area, porosity and possibly some remaining hydrophilic AL lignin. The two fractionated samples presented the highest water absorption after PLA/AL, due to higher surface rugosity, voids and increased interface area between lignin and PLA as seen with FE-SEM. The interface between PLA and other components generally increases the water absorption capability. PLA/AL-Acet and PLA/KL-Acet had lower water absorption capacity due to the reduced amount of hydroxyl groups and higher compatibility between the phases, in addition, both films had a flat surface.

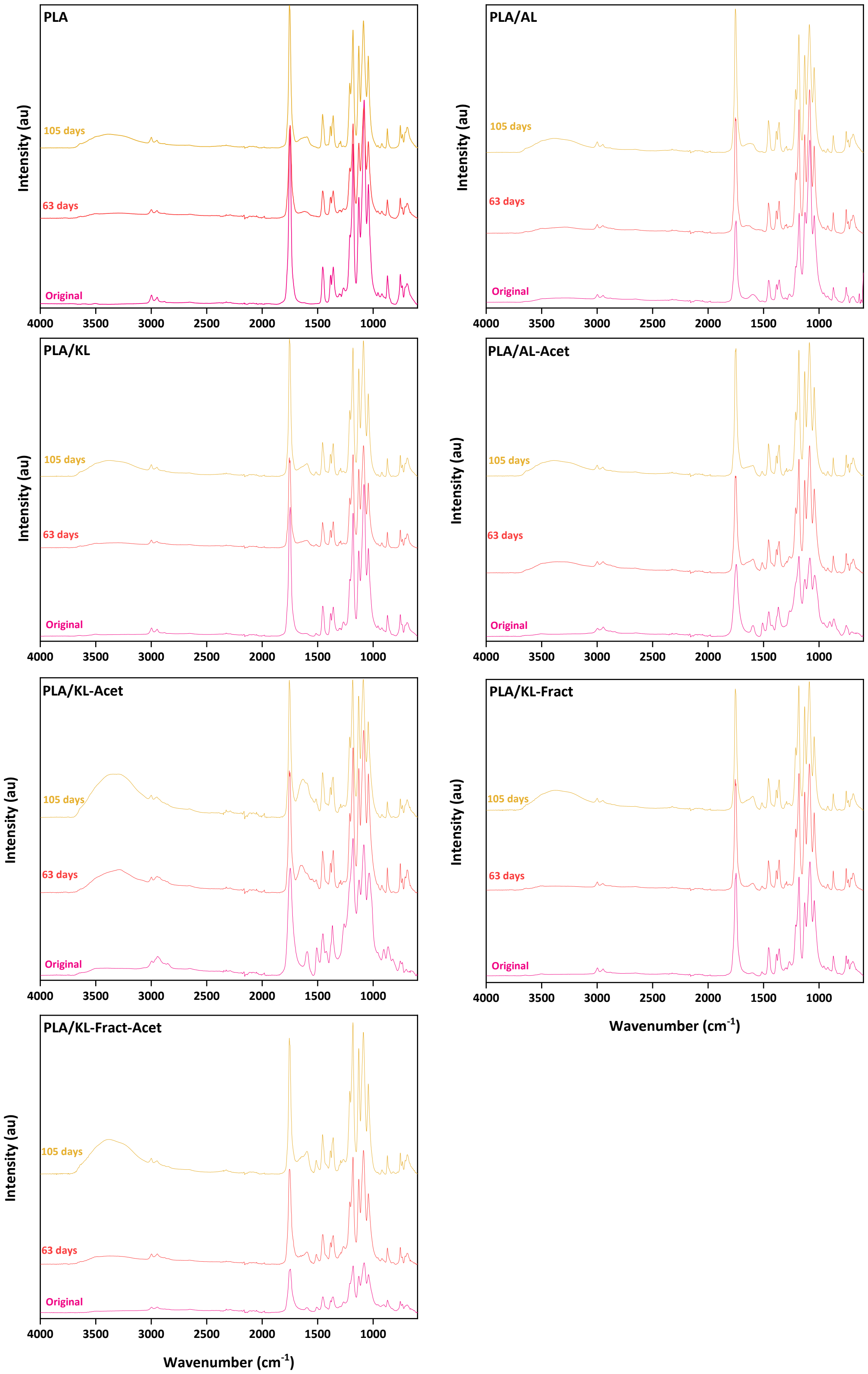

Figure S31. Evolution of functional groups of original and aged PLA and PLA/lignin films during simulated industrial composting characterized with FTIR.

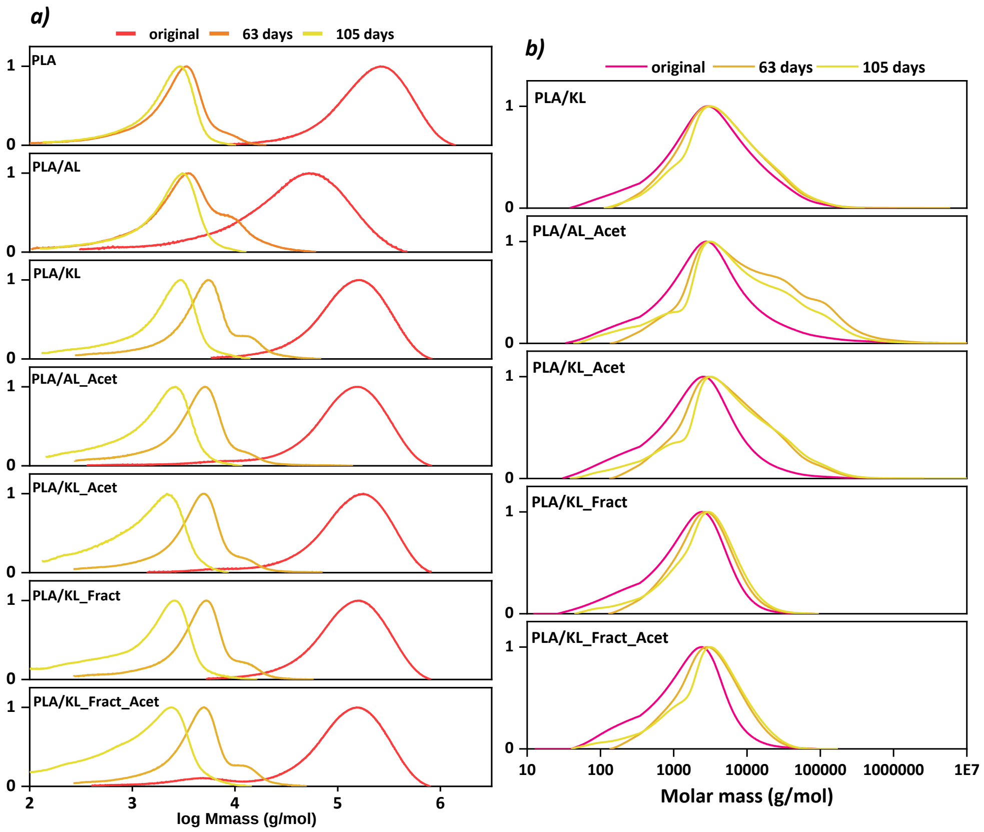

Figure S32. Evolution of molecular weight distributions during simulated composting of a) chloroform soluble fraction of PLA and PLA/lignin films and b) DMSO/LiBr soluble fractions of PLA/lignin films

References

(1) Yao, J.; Odelius, K.; Hakkarainen, M. Microwave Hydrophobized Lignin with Antioxidant Activity for Fused Filament Fabrication. *ACS Appl. Polym. Mater.* **2021**, *3* (7), 3538–3548. https://doi.org/10.1021/acsapm.1c00438

(2) Gordobil, O.; Egüés, I.; Llano-Ponte, R.; Labidi, J. Physicochemical Properties of PLA Lignin Blends. *Polym. Degrad. Stab.* **2014**, *108*, 330–338. https://doi.org/10.1016/j.polymdegradstab.2014.01.002

(3) Ainali, N. M.; Tarani, E.; Zamboulis, A.; Črešnar, K. P.; Zemljič, L. F.; Chrissafis, K.; Lambropoulou, D. A.; Bikiaris, D. N. Thermal Stability and Decomposition Mechanism of Pla Nanocomposites with Kraft Lignin and Tannin. *Polymers* **2021**, *13* (16). https://doi.org/10.3390/polym13162818.

(4) Mimini, V.; Sykacek, E.; Hashim, S. N. A.; Holzweber, J.; Hettegger, H.; Fackler, K.; Potthast, A.; Mundigler, N.; Rosenau, T. Compatibility of Kraft Lignin, Organosolv Lignin and Lignosulfonate with PLA in 3D Printing. *J. Wood Chem. Technol.* **2019**, *39* (1), 14–30. https://doi.org/10.1080/02773813.2018.1488875.

(5) Hossain, M. M.; Lokasani, V. R. Improving the Hydrophobicity of Polymers through Surface Texturing. *Annual Technical Conference - ANTEC, Conference Proceedings* **2021**, *2021-May*, 268–274.

(6) Pajer, N.; Cestari, C.; Argyropoulos, D. S.; Crestini, C. From Lignin Self Assembly to Nanoparticles Nucleation and Growth: A Critical Perspective. *npj Materials Sustainability* **2024**, *2* (1), 1–9. https://doi.org/10.1038/s44296-024-00037-5.

(7) Ji, L.; Liu, L. Y.; Cho, M.; Karaaslan, M. A.; Renneckar, S. Revisiting the Molecular weight and Conformation of Derivatized Fractionated Softwood Kraft Lignin. *Biomacromolecules* **2022**, *23* (3), 708–719. https://doi.org/10.1021/acs.biomac.1c01101.
